# An unappreciated role for neutrophil-DC hybrids in immunity to invasive fungal infections

**DOI:** 10.1371/journal.ppat.1007073

**Published:** 2018-05-21

**Authors:** J. Scott Fites, Michael Gui, John F. Kernien, Paige Negoro, Zeina Dagher, David B. Sykes, Jeniel E. Nett, Michael K. Mansour, Bruce S. Klein

**Affiliations:** 1 Department of Pediatrics, University of Wisconsin School of Medicine and Public Health, Madison, Wisconsin, United States of America; 2 Department of Medical Microbiology and Immunology, University of Wisconsin School of Medicine and Public Health, Madison, Wisconsin, United States of America; 3 Department of Medicine, University of Wisconsin School of Medicine and Public Health, Madison, Wisconsin, United States of America; 4 Division of Infectious Diseases, Department of Medicine, Massachusetts General Hospital, Boston, Massachusetts, United States of America; 5 Center for Regenerative Medicine, Massachusetts General Hospital, Boston, Massachusetts, United States of America; 6 Department of Medicine, Harvard Medical School, Boston, Massachusetts, United States of America; Rutgers New Jersey Medical School, UNITED STATES

## Abstract

Neutrophils are classically defined as terminally differentiated, short-lived cells; however, neutrophils can be long-lived with phenotypic plasticity. During inflammation, a subset of neutrophils transdifferentiate into a population called neutrophil-DC hybrids (PMN-DCs) having properties of both neutrophils and dendritic cells. While these cells ubiquitously appear during inflammation, the role of PMN-DCs in disease remains poorly understood. We observed the differentiation of PMN-DCs in pre-clinical murine models of fungal infection: blastomycosis, aspergillosis and candidiasis. Using reporter strains of fungal viability, we found that PMN-DCs associate with fungal cells and kill them more efficiently than undifferentiated canonical neutrophils. During pulmonary blastomycosis, PMN-DCs comprised less than 1% of leukocytes yet contributed up to 15% of the fungal killing. PMN-DCs displayed higher expression of pattern recognition receptors, greater phagocytosis, and heightened production of reactive oxygen species compared to canonical neutrophils. PMN-DCs also displayed prominent NETosis. To further study PMN-DC function, we exploited a granulocyte/macrophage progenitor (GMP) cell line, generated PMN-DCs to over 90% purity, and used them for adoptive transfer and antigen presentation studies. Adoptively transferred PMN-DCs from the GMP line enhanced protection against systemic infection *in vivo*. PMN-DCs pulsed with antigen activated fungal calnexin-specific transgenic T cells *in vitro* and *in vivo*, promoting the production of interferon-γ and interleukin-17 in these CD4+ T cells. Through direct fungal killing and induction of adaptive immunity, PMN-DCs are potent effectors of antifungal immunity and thereby represent innovative cell therapeutic targets in treating life-threatening fungal infections.

## Author summary

Several patient populations including those with cancer or that receive organ-transplants are at risk of life-threatening invasive fungal infections, in part due to reduced function or numbers of white blood cells. Because of limitations in antifungal drug therapy, immune-based strategies to augment white blood cells are desired to treat fungal infections. Enhancing neutrophil immunity is one important therapeutic approach to treating deadly fungal diseases. We describe a role for a poorly understood neutrophil called the neutrophil-dendritic cell hybrid (PMN-DCs) in antifungal immunity. PMN-DCs retain the microbicidal function of neutrophils, while also acquiring the capacity of dendritic cells to stimulate adaptive immunity. We show that PMN-DCs trigger adaptive immunity against fungi and are potent killers of fungal pathogens. We investigated direct killing of medically relevant fungal pathogens by PMN-DCs in preclinical mouse models and by deriving PMN-DCs from a novel neutrophil cell line. We observed that PMN-DCs killed fungal cells better than typical neutrophils. We also demonstrated that administration of PMN-DCs during systemic infection reduced fungal burden. Because PMN-DCs are such potent killers of fungal cells and concomitantly induce long-term protective immunity, these cells are important targets for immunotherapies designed to treat life-threatening fungal infections.

## Introduction

In the waging battles between host immunity and microbial invaders, polymorphonuclear cells (PMN) or neutrophils are the most numerous cellular soldiers under the host banner. Neutrophils ward off and eliminate many infections, particularly those caused by fungi. As the infantry of an inflammatory immune response, neutrophils are often the first leukocytes to infiltrate infected tissue armed with an arsenal of antimicrobial agents and functions for direct combat against intruders [1]. Classically defined, neutrophils are terminally differentiated and short-lived at sites of infection; however, there is a growing appreciation of long-lived neutrophils often accompanied by morphological changes [2]. One of these long-lived neutrophil populations has the morphology of dendritic cells (DCs) and can present antigen to T cells [3]. These neutrophils, given a variety of names including antigen-presenting neutrophils, have been characterized as a *bona fide* neutrophil population under the name neutrophil-DC hybrids since they possess properties of both neutrophils and DCs [4]. We refer to them here as PMN-DCs.

In a series of studies, highly purified murine neutrophils were shown to differentiate *in vivo* and *in vitro* into cells expressing major histocompatibility complex (MHC) class II, CD11c, and other DC markers while retaining neutrophil surface markers [4–6]. These PMN-DCs have the morphology of DCs, present antigen and promote T cell polarization, while remaining highly phagocytic cells that produce antimicrobials and release neutrophil extracellular traps (NETs).

PMN-DCs have been observed in humans and mice for decades under a variety of inflammatory conditions. In mice, PMN-DCs are characterized as being MHC class II^+^ and CD11c^+^, and in humans PMN-DCs express HLA-DR (MHC class II) with differing reports of other DC markers such as CD40, CD86 and CCR6 [3,4]. PMN-DCs arise under auto-inflammatory conditions such as Wegener’s granulomatous disease, rheumatoid arthritis and in a mouse model of inflammatory bowel disease [7–9]. In patients with bacterial infection and leishmaniasis, PMN-DCs have been found in the circulation [10–11]. HLA-DR^+^ neutrophils have also been found in tumors of lung cancer patients [12]. Adjunctive therapy with GM-CSF (granulocyte-macrophage colony stimulating factor) or interferon (IFN)-γ also induces the appearance of PMN-DCs in human patients [13,14]. While being observed under a variety of inflammatory circumstances, the role that PMN-DCs play–helpful or harmful—in disease is poorly understood. Because PMN-DCs retain neutrophil microbicidal functions and can polarize inflammatory T cell functions [4], we hypothesize that PMN-DCs aid in immunity to infections where neutrophils and T helper (Th) type 1 and 17 responses are important e.g. fungal infections [15].

Neutrophils are essential in immunity to many fungal infections including the common causes of serious and lethal fungal diseases, *Aspergillus* and *Candida* species [16,17]. Candidemia is the 4^th^ most common nosocomial bloodstream infection in the U.S., with a mortality rate of 43% [18,19]. Mortality rates of invasive aspergillosis also exceed 40% [20]. Persons at greatest risk of serious fungal infections by these and other fungi are patients with cancer or solid organ transplant, often due to deficiencies in neutrophil immunity [16]. In heart and lung transplant recipients, incidence rates of invasive fungal infections are as high as 25%, with 90-day mortality rates of 23% [21,22]. In patients with hematological malignancies, rates of serious fungal infection reach 18%, and mortality rates, 20–30% [23–25].

Most serious fungal infections are treated with antifungal drugs, but antifungals can be ineffective due to fungal resistance, low bioavailability, toxicity, and limited spectrum of activity [26]. Due to these limits, immunotherapies are needed. No commercial vaccine against fungi is available, but some studies have investigated vaccine prevention against fungal disease [27,28]. Chimeric antigen receptor-modified T cells, a novel therapy for cancer patients, may be effective in treating fungal infections [29,30]. Neutrophil-directed therapies have been used to prevent and treat fungal infections, particularly in patients with neutropenia [31]. Ideally, immunotherapies against fungal infections should enhance innate killing mechanisms or promote long-term adaptive immunity. Thus, any therapies that target PMN-DCs would be promising because these cells enact both innate and adaptive immunity.

To study the emergence and function of PMN-DCs during fungal infection, we initially analyzed neutrophils in a murine model of pulmonary blastomycosis, a fungal pneumonia. Blastomycosis is a pyogranulomatous disease eliciting exuberant, sustained recruitment of neutrophils [32,33], permitting analysis of small neutrophil populations. Blastomycosis, like aspergillosis, is usually initiated by inhalation of spores from the environment [34].

Herein, we report that during pulmonary blastomycosis, PMN-DCs expanded and associated with *Blastomyces dermatitidis* more frequently than any other leukocyte. PMN-DCs also killed yeast better than undifferentiated canonical neutrophils. We extended our observations to other common human fungal infections, aspergillosis and candidiasis, finding that PMN-DCs emerge in murine models of these infections and kill these fungi better than canonical neutrophils. Additionally, we exploited a neutrophil progenitor cell line to generate a highly pure population of PMN-DCs without any taxing enrichment procedure. Adoptive transfer of PMN-DCs, derived from the cell line, reduced fungal burden during murine system candidiasis. We also used these cell line PMN-DCs to demonstrate that PMN-DCs efficiently present fungal antigen and prime protective Th1 and Th17 responses. In sum, PMN-DCs expand to a small proportion of neutrophils during infection, but they associate with and kill fungal cells far better than canonical neutrophils.

Due to the longevity of PMN-DCs and their ability to prime adaptive responses, we believe that these cells contribute significantly to antifungal immunity and can be harnessed therapeutically as potent mediators of protection.

## Results

### Expansion of neutrophils with DC markers and morphology during pulmonary blastomycosis

**Fig 1:**
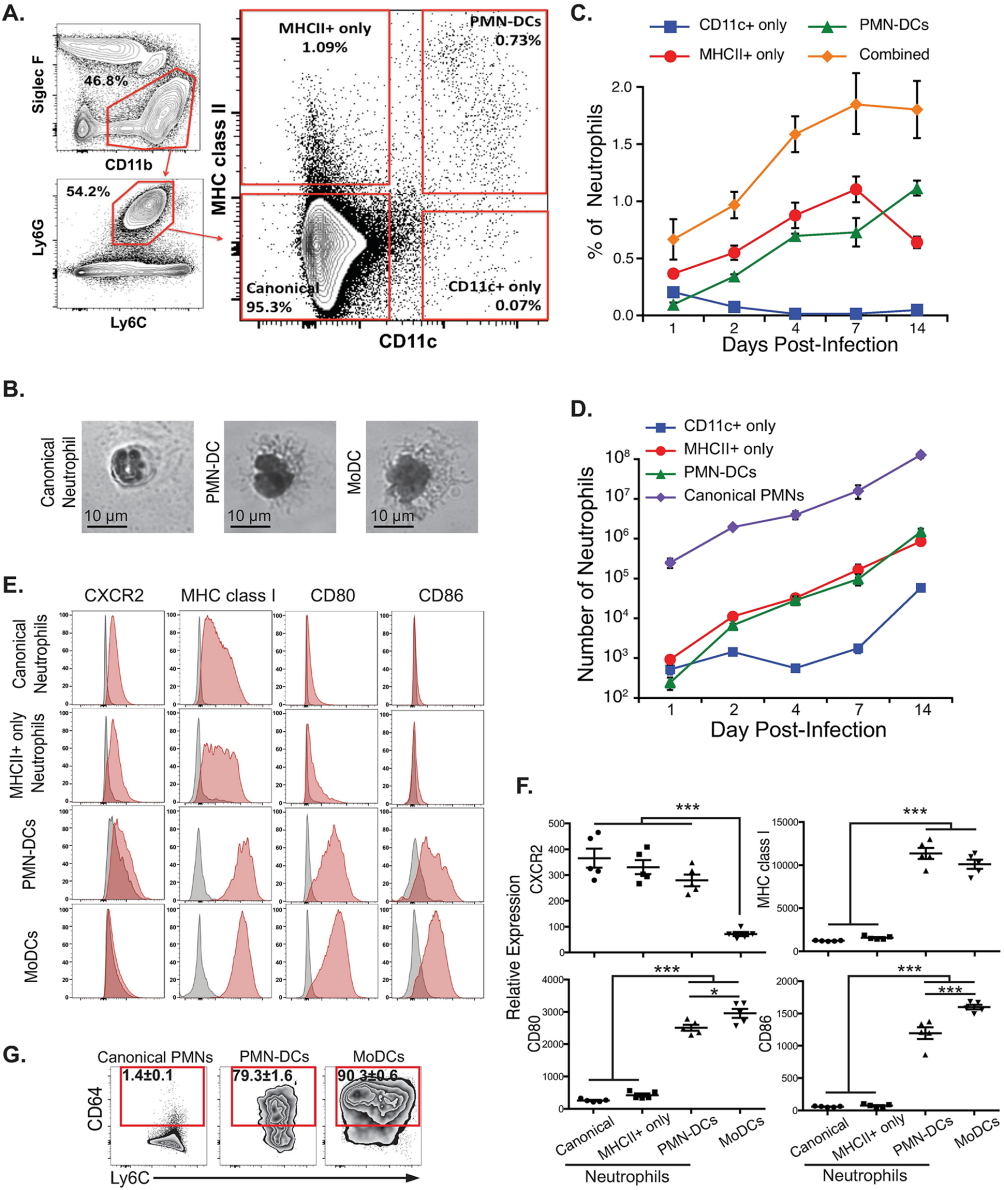
Neutrophils with DC characteristics (PMN-DCs) emerge during pulmonary blastomycosis. (**A**) Neutrophil gating strategy showing representative lung sample 7 dpi with *Blastomyces*. (**B**) Canonical neutrophils (CD11b^+^, Ly6G^+^, Ly6C^int^, CD11c^-^), PMN-DCs (CD11b^+^, Ly6G^+^, Ly6C^int^, CD11c^+^), and MoDCs (CD11b^+^, Ly6G^-^, Ly6C^+^, CD11c^+^) were FACS sorted from *Blastomyces*-infected lungs at day 7 then stained with a Hema3 kit for microscopy. (**C-D**) Kinetic analysis of neutrophil differentiation after intratracheal challenge with *Blastomyces* showing relative proportion (C) and absolute numbers (D) of differentiated neutrophils (parent CD11b^+^, Ly6G^+^, Ly6C^int^); the combined population is the sum of all neutrophils expressing either CD11c or MHC class II. (**E-F**) Surface expression of neutrophil marker (CXCR2) and antigen-presenting cell markers (MHC class I, CD80, CD86) on neutrophil populations and moDCs (S1C Fig) indicated by histograms (E) and relative expression (F) as indicated by mean fluorescence over fluorescence minus one (FMO) control (gray histogram). (**G**) Expression of CD64 on neutrophil and MoDC populations. Representative experiments shown; mean±SEM indicated; N = 3–5 mice/group; C-D show representative experiment from 3 kinetic experiments; E-G show representative results from at least two independent experiments at 7 dpi with *Blastomyces*.

Of the copious neutrophils in the blastomycotic lung [32,33], one population is phenotypically converted into PMN-DCs (Fig 1A and 1B). PMN-DCs were absent from the lungs of naïve mice, but emerged after neutrophils extravasated across the lung capillaries (Fig 1C and 1D, S1A Fig). The proportion of neutrophils that converted phenotypically to PMN-DCs rose rapidly after one day and steadily increased over 14 days (Fig 1C) until mice succumbed of pneumonia. The absolute number of PMN-DCs in the lung approached 10^5^ after one week and 10^6^ after two weeks (Fig 1D) making this population large enough to analyze in this pre-clinical model.

PMN-DCs that appeared during pulmonary blastomycosis had DC morphology (Fig 1B, S1B–S1D Fig) and expressed DC markers (MHC class II, CD11c, CD80, CD86, CD40) while retaining neutrophil levels of expression of Ly6G, Ly6C, CD11b, and CXCR2 (Fig 1A, 1E and 1F, S2 Fig). The lack of Siglec F, NK1.1, and F4/80 expression also indicated that the PMN-DCs observed were not contaminating populations of macrophages or natural killer cells (Fig 1A, S2C Fig). Overall, these PMN-DCs resembled the characterized neutrophil-DC hybrids in mice [3–5]. Also, as previously characterized [3,4], PMN-DCs appear to be neutrophil-derived and not monocyte-derived because they express Ly6G, a unique marker of murine neutrophils, and do not express CD115 (macrophage-colony stimulating factor receptor) found on monocytes and monocyte-derived cells (S3a Fig). Additionally, equivalent numbers of PMN-DCs appeared in wild-type mice and *ccr2*^*-/-*^ mice with severe defects in monocyte recruitment (S3b Fig) [35].

In mice, PMN-DCs express CD11c and MHC class II [4]. In humans, these neutrophils are defined by expression of HLA-DR (MHC class II), although other DC markers are expressed [3]. We tracked all neutrophils that expressed either MHC class II or CD11c during pulmonary blastomycosis. CD11c^+^ only (MHCII^-^ CD11c^+^) neutrophils were virtually absent, decreasing in proportion from a minuscule population after day 1 (Fig 1C). MHC class II^+^ only (MHCII^+^CD11c^-^) neutrophils and PMN-DCs (MHCII^+^ CD11c^+^) rose in proportion through the first week. MHC class II^+^ only neutrophils showed lower expression of MHC class II than did PMN-DCs (Fig 1A, S2A Fig), suggesting that this population may be intermediate between classical neutrophils and PMN-DCs. PMN-DCs expressed MHC class I and co-stimulatory ligands CD80 and CD86 similar to levels expressed by DCs (Fig 1E and 1F). MHCII^+^ only neutrophils had low expression of DC markers, further indicating that they may be intermediately differentiated cells. Additionally, both PMN-DCs and MHC class II^+^ only neutrophils increased together through the first 7 days of infection and the relative proportions flipped at day 14 when the number of PMN-DCs surpassed MHC class II^+^ only neutrophils (Fig 1C and 1D).

One unanswered question about the biology of PMN-DCs concerns the type of DC that PMN-DCs most resemble. PMN-DCs, differentiated *in vitro*, are transcriptionally similar to DCs differentiated from bone marrow monocytes [4]. We examined the expression of surface markers that distinguish DC subsets [36]. Not surprisingly, PMN-DCs during early blastomycosis did not express CD8a or langerin found on DCs in lymphatic and skin tissue, respectively (S4A Fig). PMN-DCs also did not express CD103, which is found on a population of resident DCs (S4A Fig). Nor did PMN-DCs express the plasmacytoid DC (pDC) markers B220 or Siglec H; however, a proportion of the MHCII^+^ only neutrophils did express B220, but not Siglec H, indicating that some cells in this population may be a neutrophil that resembles pDCs (S4C and S4D Fig). The monomeric Fc γ receptor CD64, expressed highly on inflammatory monocyte-derived DCs (moDCs) [37] was expressed by a majority of PMN-DCs (Fig 1G, S4B Fig). These data support the idea that PMN-DCs are most like moDCs.

### A role for PMN-DCs in fungal clearance

**Fig 2:**
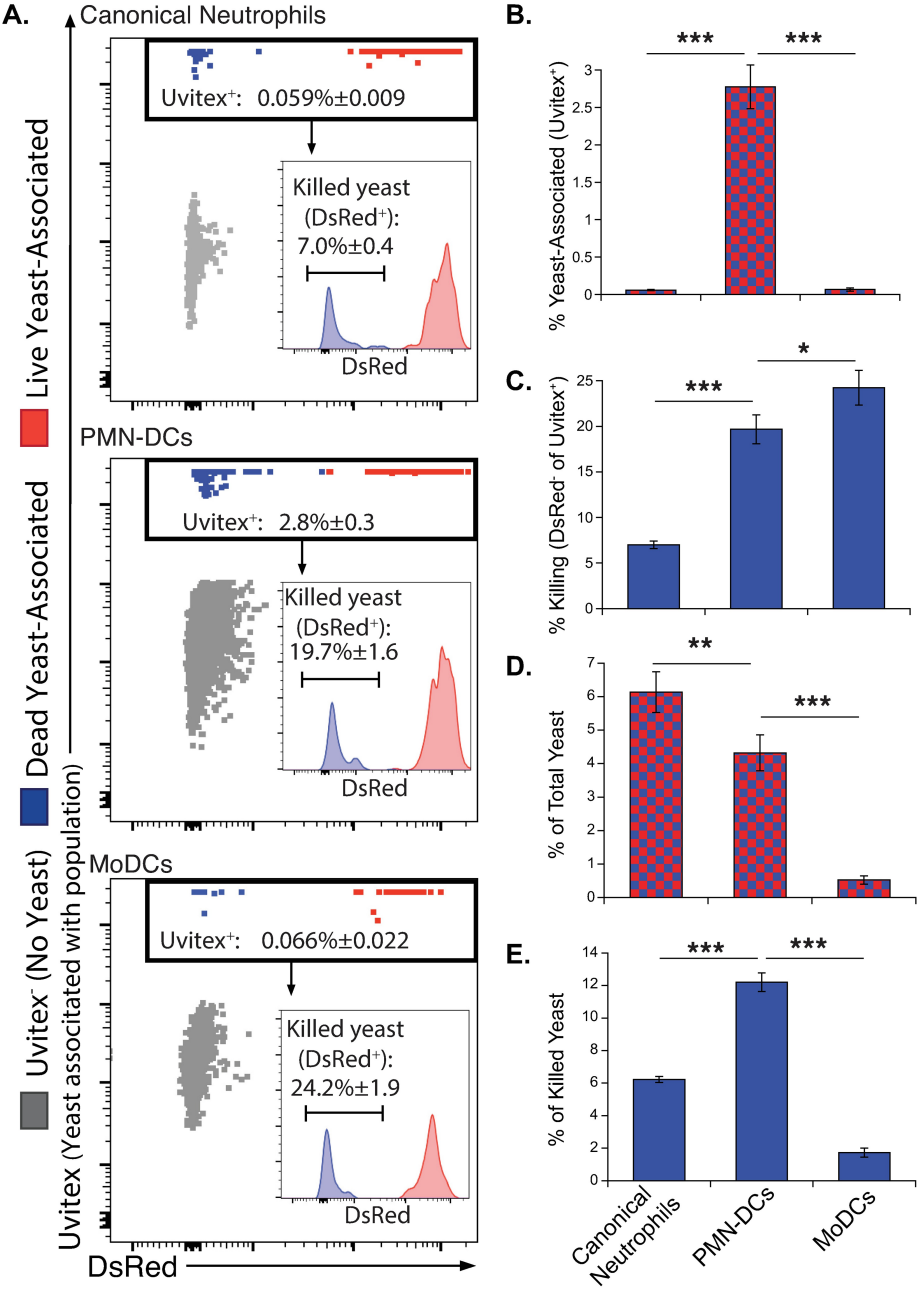
PMN-DCs associate with yeast and kill yeast at higher frequencies than canonical neutrophils. Mice were challenged with DsRed *B*. *dermatitidis*, and lungs were harvested at 7 dpi. (**A**) Representative plots showing canonical neutrophils, PMN-DCs and moDCs indicating the cells of each population associated with yeast (% Uvitex^+^). Inset histograms show Uvitex^+^ events indicating live (DsRed^+^) and dead (DsRed^-^, % noted) yeast associated with each population. (**B**) The proportion of each population associated with yeast as indicated by Uvitex staining. (**C**) The killing rate–proportion of Uvitex^+^ yeast that are DsRed^-^. (**D**) The proportion of total yeast (all Uvitex^+^ events) associated with indicated leukocytes. (**E**) The contribution to yeast killing is indicated by the proportion of total killed yeast in the lung (all DsRed^-^Uvitex^+^ events) associated with each population. Panel A shows a single representative experiment of 4 independent experiments; N = 5 mice; means ± SEM indicated.

While the appearance of PMN-DCs during inflammation and the various functions of PMN-DCs have been documented [3], the role of these cells during disease is poorly understood. Because PMN-DCs retain microbicidal properties of neutrophils, we investigated the role of PMN-DCs in killing fungi during infection. We previously created a DsRed strain of *Blastomyces* that reports yeast viability e.g. when yeasts are killed they lose DsRed fluorescence [35]. In tandem with Uvitex cell wall stain, we tracked live and killed yeast associated with leukocyte populations during infection (Fig 2A). At 7 days post-infection (dpi), when a relatively large PMN-DC population expanded, canonical neutrophils had low association rates with yeast (<0.1%), whereas PMN-DCs had 30-fold higher association rates, showing up to 3% of PMN-DCs associated with yeast (Fig 2A and 2B). PMN-DCs also killed yeast at twice the rate of canonical neutrophils (Fig 2A and 2C). Overall, despite amounting to less than 1% of the lung leukocytes during early infection (Fig 1), PMN-DCs associated with ≈5% of the total yeast in the lung (Fig 2D) and accounted for up to 15% of the killed yeast in the lung (Fig 2E).

Previously, we observed that monocytes and moDCs had high rates of killing but a low association with *B*. *dermatitidis in vivo* [35]. Similarly, we saw that moDCs had a higher rate of killing compared to PMN-DCs, but much lower association with yeast (Fig 2).

**Fig 3:**
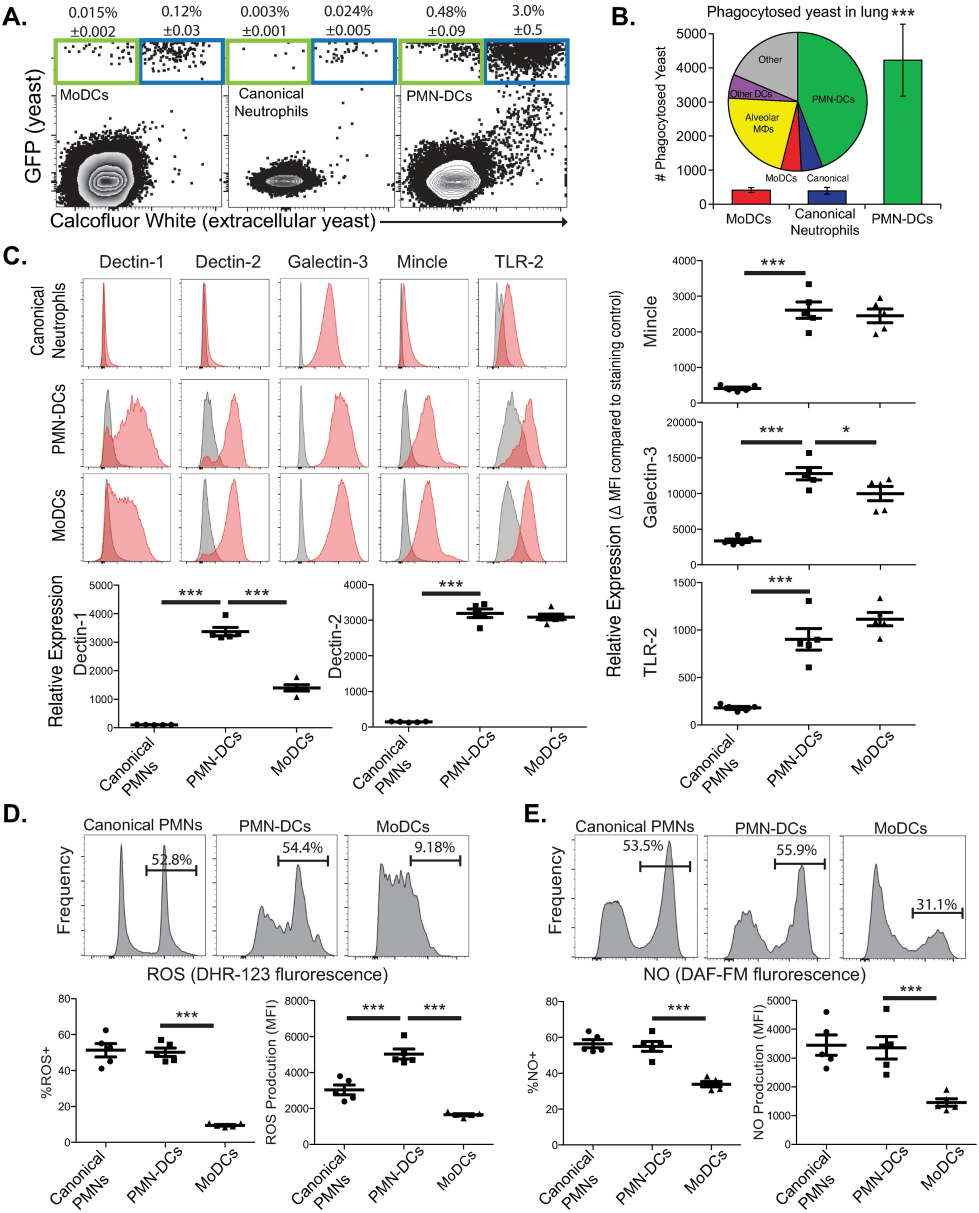
Phagocyte functions of PMN-DCs. (**A-B**) Mice were challenged with GFP *B*. *dermatitidis*, and lungs were harvested 7 dpi and stained with calcofluor white to mark extracellular yeast. (A) Representative flow plots showing association of gated leukocyte populations with extracellular (Calcfluor^+^, blue) and phagocytosed (Calcofluor^-^, green) yeast (GFP^+^). (B) Absolute number (histogram bars) of phagocytosed yeast by moDCs, canonical neutrophils or PMN-DCs with inset pie chart indicating the proportion of all yeast phagocytosis by leukocyte populations in the lung. (**C**) Surface expression of fungal-recognizing pattern recognition receptors on neutrophil populations and moDCs at 7 dpi with *B*. *dermatitidis*. Histograms show FMO controls (gray) and stained populations (red); relative expression of each receptor on each population. (**E**–**F**) *Ex vivo* staining of neutrophil populations and moDCs with ROS-indicator DHR-123 (C) or NO-indicator DAF-FM (D). Representative histograms shown on top; proportions of ROS^+^ and NO^+^ on the bottom left and ROS/NO production indicate by MFI on the right. All experiments are representative of at least two independent experiments. N = 5 mice. Means ± SEM indicated.

We tracked killing of yeast at an earlier time point, 2 dpi, when PMN-DCs were less established. Even earlier in infection PMN-DCs still had higher association rates with yeast and killed yeast better than did canonical neutrophils (S5A–S5D Fig). Because uvitex staining persists on fungi for about 2 days *in vivo*, we stained yeast before infection allowing us to confirm that the high association rate of PMN-DCs with yeast was not a confounding effect of non-specific intracellular Uvitex staining. Separately, we tracked association of GFP yeast with PMN-DCs at 7 dpi and found comparable results (Fig 3A).

We considered the possibility that, *in vivo*, PMN-DCs engulf yeast already killed by neutrophils or other cells. To exclude this possibility, we FACS-sorted canonical neutrophils, PMN-DCs and moDCs from cultured bone marrow leukocytes and incubated these cells with *Blastomyces* yeast to investigate direct killing by PMN-DCs. *In vitro*, sorted PMN-DCs killed yeast better than sorted canonical neutrophils (S5E Fig), but less well than moDCs, reproducing *in vivo* results. Taken together, these data indicate that PMN-DCs represent a significant leukocyte effector during lethal fungal pneumonia due to blastomycosis.

### Mechanisms of PMN-DC killing of fungal cells

Because yeasts were more frequently associated with PMN-DCs than classical neutrophils during infection, we asked whether PMN-DCs phagocytose yeast better than neutrophils. To test this, we challenged mice with GFP-expressing *B*. *dermatitidis* yeast and surfaced-stained samples with calcofluor white, a chitin cell-wall stain, to distinguish extracellular yeast (Calcofluor^+^) from intracellular yeast (Calcofluor^-^) (Calcofluor is not cell membrane permeable). PMN-DCs associated much more frequently with Calcofluor^-^ yeast than did canonical neutrophils. About 15% of PMN-DCs associated with yeast had phagocytosed the yeast (Fig 3A). PMN-DCs phagocytosed 8-fold more yeasts than canonical neutrophils or moDCs, and around 40% of all phagocytosed yeasts in the lung were engulfed by PMN-DCs (Fig 3B).

We quantified the expression of pattern recognition receptors (PRR) on the surface of PMN-DCs during early blastomycosis to discern what may explain the higher association and phagocytosis rates by PMN-DCs. Dectin-1, Dectin-2, Mincle, mannose receptor, TLR-2, and TLR-4 are important surface receptors that recognize fungal pathogen-associated molecular patterns [15]; of these, Dectin-2 is essential for recognizing *Blastomyces* [38]. We found that PMN-DCs appearing in response to *Blastomyces* had higher expression of these PRRs on their surface than canonical neutrophils (Fig 3C, S6 Fig). Surface expression of Dectin-2, Mincle, mannose receptor, TLR-2 and TLR-4 on PMN-DCs was equivalent to that on moDCs; however, PMN-DCs had even greater surface expression of Dectin-1 and Galectin-3 than did moDCs.

Reactive oxygen species (ROS) and nitric oxide (NO) are important killing mechanisms of *B*. *dermatitidis* and other fungi [16,35,39,40]. To identify the mechanism of fungal killing by PMN-DCs, we quantified ROS and NO production during blastomycosis. We stained leukocytes *ex vivo* with dihydrorhodamine-123 and DAF-FM, fluorescent indicators of ROS and NO respectively [41]. While similar proportions of PMN-DCs and canonical neutrophils produced ROS, PMN-DCs produced more ROS than canonical neutrophils (Fig 3D). PMN-DCs also produced more ROS than canonical neutrophils when stimulated *ex vivo* with f-MLP, a potent inducer of neutrophil ROS (S7A Fig). Neutrophils produced significantly more NO than moDCs, but PMN-DCs and canonical neutrophils produced equivalent NO with comparable proportions of NO^+^ cells (Fig 3E). We also asked whether PMN-DCs could be induced to produce more NO by stimulating them *ex vivo* with LPS; we saw no enhancement of NO production by PMN-DCs versus canonical neutrophils under these conditions (S7B Fig).

These data indicate that PMN-DCs retain neutrophil functions of phagocytosis and production of ROS and NO necessary to kill fungal cells. PMN-DCs also display greater surface expression of PRRs than do canonical neutrophils, possibly contributing to the enhanced fungal killing. The high association rate of PMN-DCs with yeast, combined with antimicrobial defenses that are as good or better than canonical neutrophils, underscore that PMN-DCs are significant effectors of immunity to blastomycotic pneumonia.

### PMN-DCs in candidiasis and aspergillosis

Because PMN-DCs contribute significantly to yeast killing during pulmonary blastomycosis, we asked whether they emerge during other fungal infections and contribute to killing. Neutrophil immunity is essential for clearing *Aspergillus* and *Candida* infections [16]. If the immunity enacted by PMN-DCs endures for these systemic fungal infections, PMN-DCs would be an important adjunct for cellular immunotherapy against these infections.

**Fig 4:**
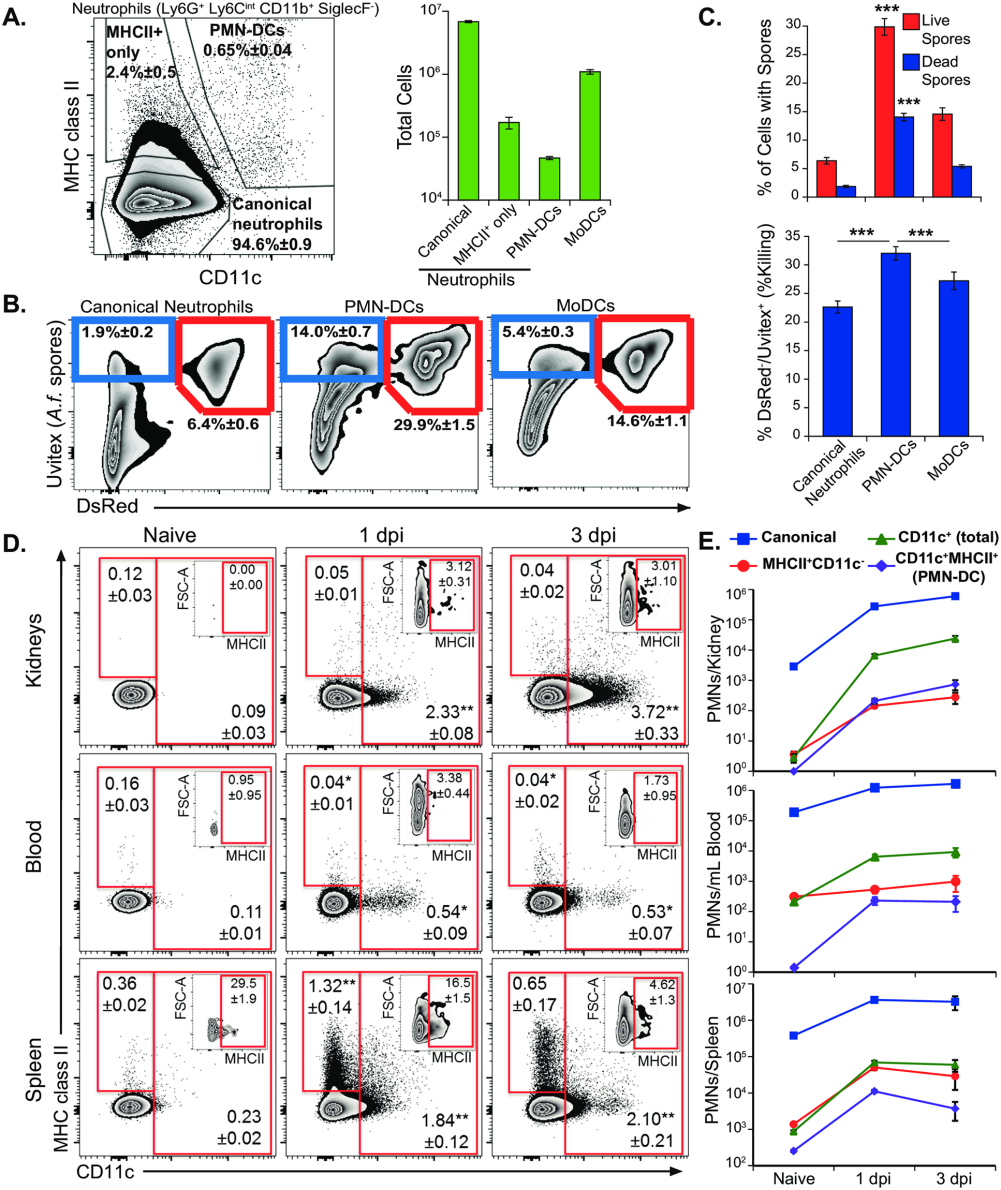
Appearance of PMN-DCs and fungal killing by the cells during pulmonary aspergillosis and systemic candidiasis. (**A**–**C**) Mice were infected IT with Uvitex-stained DsRed *A*. *fumigatus* spores, and lungs were harvest 48 hours later. (**A**) Proportions and absolute numbers of canonical and MHCII^+^ only neutrophils, PMN-DCs (MHCII^+^ CD11c^+^) and moDCs in the lung. (**B**) Representative plots showing association of leukocyte populations with live (DsRed^+^) and killed (DsRed^-^) *Aspergillus* spores (Uvitex^+^). (**C**) Proportion of leukocytes associated with live and killed spores (top) and killing rate (% DsRed^-^/Uvitex^+^) (bottom) of spores by leukocytes in the lungs. (**D**–**E**) Mice were challenged IV with *C*. *albicans* yeast; kidneys, spleens, and peripheral blood were harvested at day 1 or 3 or from naïve mice (day 0). (**D**) Representative plots showing neutrophils (CD11b^+^, Ly6G^+^, Ly6G^int^, Siglec F^-^) with inset plots indicating the proportion of CD11c^+^ neutrophils expressing MHC class II. (**E**) Time course showing absolute numbers of canonical (MHCII^-^ CD11c^-^), MHCII^+^ CD11c^-^, CD11c^+^ and MHCII^+^ CD11c^+^ neutrophils in tissues during systemic candidiasis. All data are representative of at least three independent experiments. N = 3–5 mice. Means±SEM indicated. For C, statistical comparisons were among leukocyte populations; for D, statistical comparisons were with Day 0 control.

We infected mice with *A*. *fumigatus* spores intratracheally (IT) to see if PMN-DCs expand during this infection. *Aspergillus* spores are rapidly cleared from healthy wild-type mice, so we looked for neutrophil differentiation at 48 hours when spores are still present in the lung. PMN-DCs (MHCII^+^CD11c^+^) differentiated and comprised 0.7% of neutrophils (Fig 4A). We challenged mice with DsRed *A*. *fumigatus* [42] to track association and killing by neutrophil populations (Fig 4B). As in blastomycosis, *A*. *fumigatus* spores associated more frequently with PMN-DCs and were killed at a higher rate by PMN-DCs than canonical neutrophils (Fig 4C, S8 Fig).

PMN-DCs expand under inflammatory conditions in a variety of tissues [3,5]. We analyzed the numbers and differentiation state of neutrophils in a model of systemic candidiasis where *C*. *albicans* is administered intravenously (IV) [19]. The primary target organ of systemic candidiasis is the kidney [43]. We tracked neutrophils and their differentiation in the kidney, as well as the peripheral blood and spleen, where *Candida* appears early in infection. After challenge, CD11c expression was upregulated on neutrophils in the kidney, peripheral blood, and spleen; however, MHC class II was not greatly upregulated on neutrophils in the kidney or peripheral blood (Fig 4D). Nevertheless, *Candida* infection induced significant increases in neutrophil numbers in these tissues. With the increase in recruitment and differentiation of neutrophils, the numbers of CD11c^+^ or MHCII^+^ neutrophils rose by orders of magnitude by 24 hours post infection (Fig 4E). Thus, these data from pre-clinical models of systemic candidiasis, as well as pulmonary infection with *Aspergillus* or *Blastomyces*, identify PMN-DCs as important leukocyte effectors in several systemic fungal infections.

### Generating PMN-DCs from a neutrophil progenitor cell line

To further investigate the role of PMN-DCs in antifungal immunity, we exploited a highly enriched population of PMN-DCs. PMN-DCs have previously been differentiated from primary neutrophils, but murine neutrophils require feeder cells for *in vitro* differentiation [4,6]. To obtain a sufficient number of pure or highly enriched PMN-DCs, extensive FACS sorting, time, and cost are required. These manipulations also jeopardize the quality of the cells. To circumvent these issues, we generated PMN-DCs from a neutrophil progenitor cell line. This line is a granulocyte/macrophage progenitor (GMP) that is maintained in progenitor status in the presence of estrogen by fusing the transcription factor HoxB8 to a truncated estrogen receptor (ER), and, when removed from estrogen, ≈99% of these GMPs differentiate into neutrophils [44]. We differentiated GMPs into ≥95% neutrophils after 4–5 days of estrogen removal, and, importantly, GMPs did not differentiate down the monocyte/macrophage pathway because no cells in the neutrophil population expressed CD115 or F4/80 (S9 Fig).

**Fig 5:**
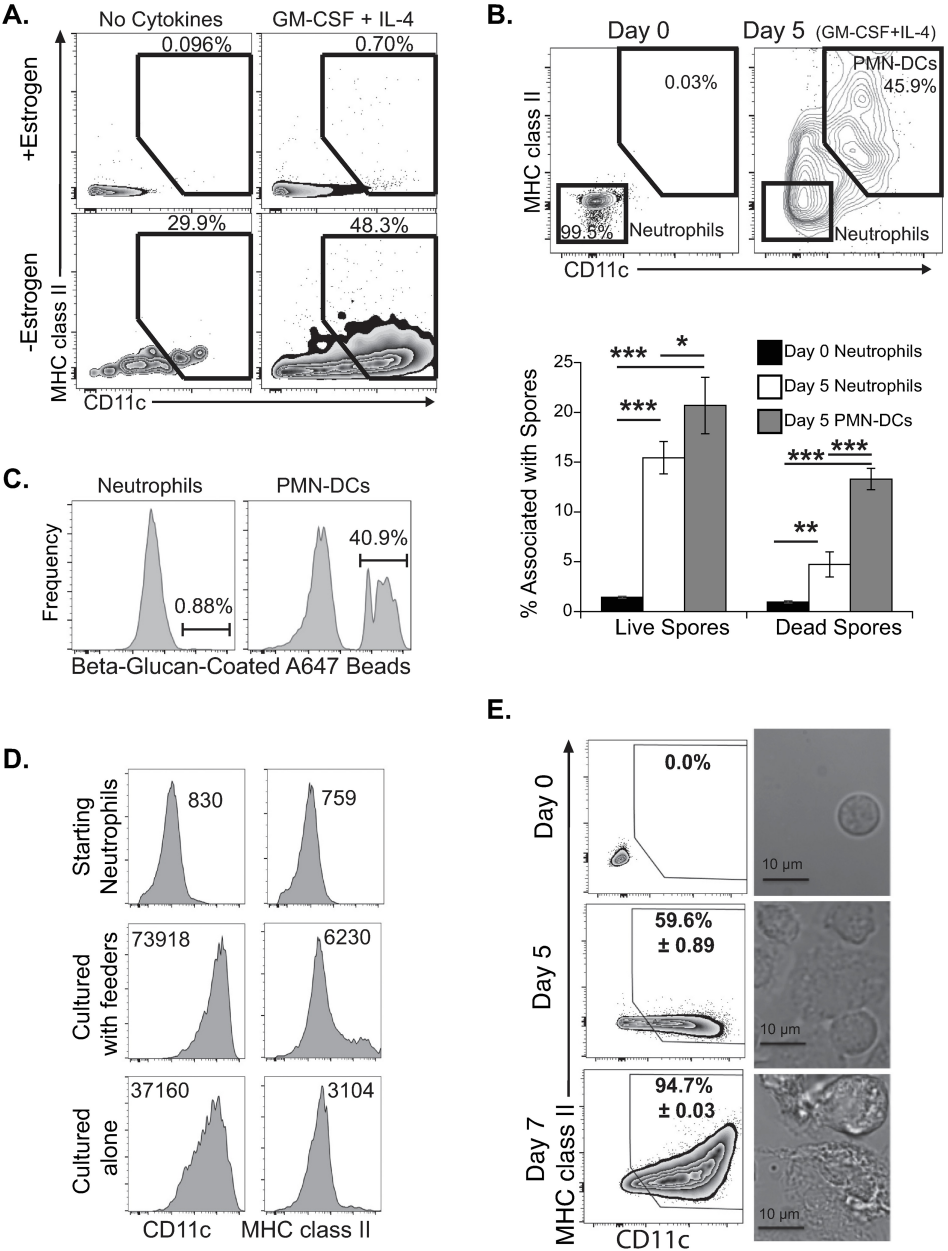
Differentiation of granulocyte/macrophage progenitor (GMP) cell line into PMN-DCs *in vitro*. (**A**) ER-HoxB8 GMP cells (GFP^+^) were cultured for 4–5 days in the presence or absence of estrogen and then cultured for an additional 5 days with or without GM-CSF or IL-4 in the presence of bone marrow feeder cells; differentiation of GFP^+^ cells into PMN-DCs was tracked by CD11c and MHC class II expression. **(B-C)** GMP cells were matured into neutrophils (Day 0) or further differentiated into PMN-DCs (after 5 days with GM-CSF and IL-4 and feeder cells). (**B**) Undifferentiated (Day 0) or differentiated (Day 5) neutrophils (upper panel) were incubated overnight with DsRed *A*. *fumigatus* spores stained with Uvitex and then analyzed by flow cytometry; the association rate with live (DsRed^+^) or dead (DsRed^-^) *A*. *fumigatus* spores with each population from above plots is shown in the lower panel. (**C**) Undifferentiated neutrophils or differentiated PMN-DCs were incubated for 1 hour with β-glucan-coated AlexaFluor647 beads and analyzed by flow cytometry for association with cells. (**D**) Expression of CD11c and MHC class II on starting neutrophils (Day 0) vs. neutrophils differentiated for 5 days with or without feeder cells (MFI indicated). (**E**) GMP cells were matured into neutrophils (Day 0) and differentiated 5–7 days without feeder cells; representative flow plots and images of cells at day 0, 5 or 7. Mean ± SEM shown.

To determine whether the ER-HoxB8 GMPs can be differentiated into PMN-DCs, we cultured GFP-expressing GMPs in the presence or absence of estrogen, then added GM-CSF and IL-4 with bone marrow feeder cells [6]. When GMPs matured into neutrophils in the absence of estrogen, a greater proportion and number of neutrophils became PMN-DCs (Fig 5A). When estrogen was maintained, the cells had no increase in CD11c or MHC class II expression. Under differentiation conditions for 5 days, nearly 50% of GFP^+^ cells expressed CD11c with some increase in MHC class II expression.

We assessed the function of GMP-derived PMN-DCs. Because primary PMN-DCs associated more frequently with fungal cells and killed them better than canonical neutrophils, we generated neutrophils and PMN-DCs and incubated them with Uvitex-stained DsRed *A*. *fumigatus* spores. Differentiated PMN-DCs associated with and killed spores at a higher rate than did undifferentiated neutrophils (day 0) or neutrophils that remained undifferentiated despite culture with cytokines (day 5) (Fig 5B, lower panel). Notably, PMN-DCs upregulated MHC class II expression after culture with spores (Fig 5B, upper panel). We also compared GMP-derived PMN-DCs and undifferentiated neutrophils in their ability to associate with or internalize fluorescent beads coated with β-1,3-glucan, a fungal cell wall glycan. After 1 hour, less than 1% of neutrophils were associated with β-glucan beads as compared to over 40% of PMN-DCs (Fig 5C).

We next optimized culture of GMP-derived neutrophils to obtain higher purities of PMN-DCs. Since removal of feeder cells is the greatest obstacle to obtaining pure murine PMN-DCs *in vitro*, we asked whether feeder cells are necessary in generating PMN-DCs from the cell line. After maturing GMP cells to neutrophils, we cultured neutrophils with or without feeder cells in the presence of GM-CSF and IL-4 for 5 days. Remarkably, we saw over 50% differentiation into PMN-DCs in the absence of feeder cells with only minor reductions in CD11c and MHC class II expression (Fig 5D and 5E). Along with induction of DC markers, we observed morphological changes in the GMP-derived neutrophils from small, round cells with a segmented nucleus to large cells with membrane projections and mononuclear phenotype (S10 Fig). To obtain a higher purity of PMN-DCs, we cultured GMP-derived neutrophils longer with GM-CSF and IL-4. After 7 days, over 90% of cells showed increased CD11c expression and greater morphological changes (Fig 5E, S10 Fig).

### Using ER-HoxB8 GMP cell line to probe PMN-DC immunity

**Fig 6:**
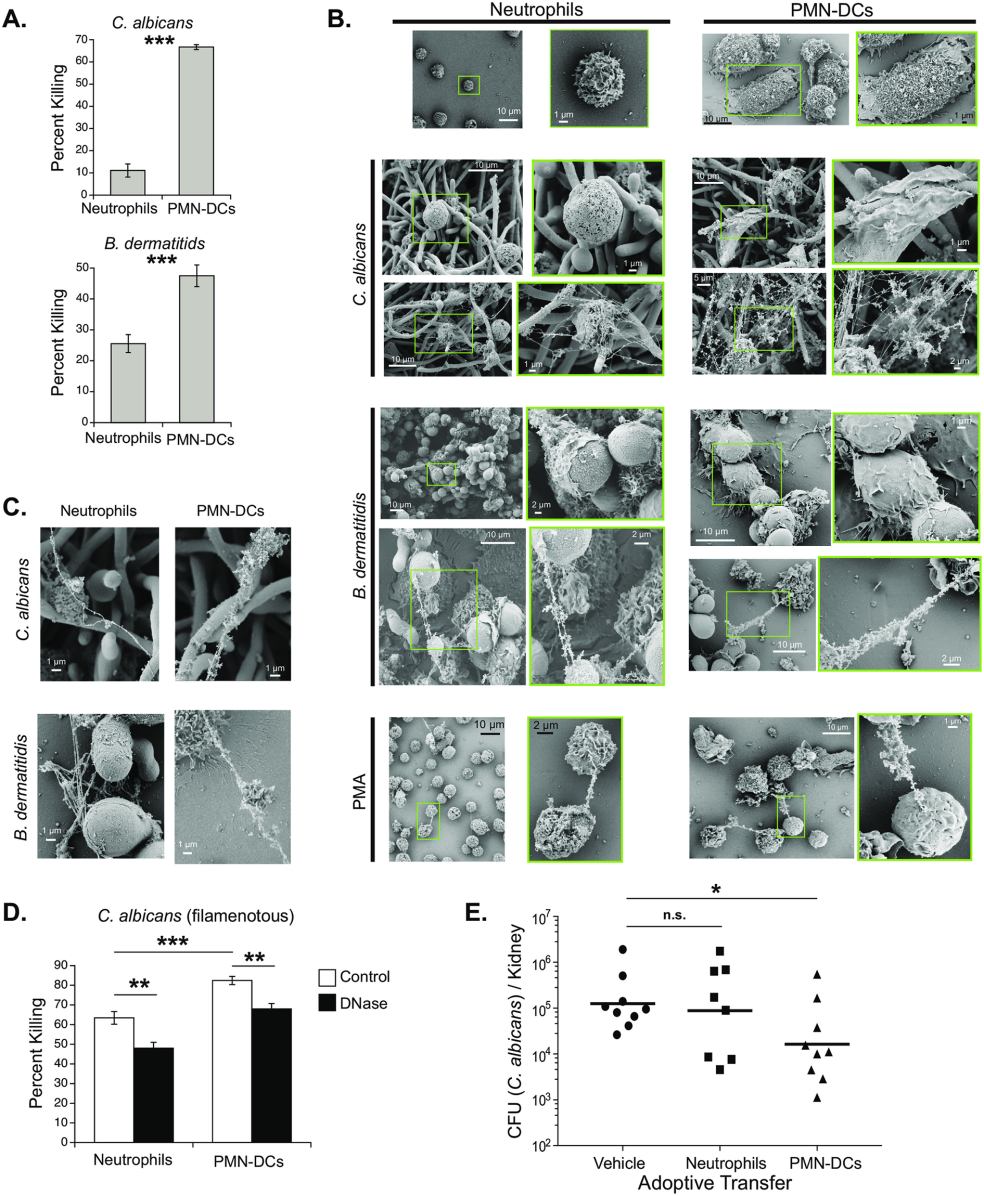
Direct fungal killing by PMN-DCs and protection by adoptive transfer of PMN-DCs. Canonical neutrophils or PMN-DCs were generated *in vitro* from the GMP cell line and co-cultured *in vitro* with fungi (A-D) or transferred IV into mice (E). (**A**) Fungal killing by canonical neutrophils or PMN-DCs during *in vitro* culture with *C*. *albicans* yeast (top) or *B*. *dermatitidis* yeast (bottom). (**B**) Scanning electron microscopy (SEM) of canonical neutrophils or PMN-DCs alone, with *C*. *albicans* or *B*. *dermatitidis*, or stimulated with PMA. Higher magnification images of highlighted boxes are shown to the right of wider image. For interactions with fungi, top images show interactions between cells and fungi highlighting phagocytosis and attempted phagocytosis; bottom images show NETs. (**C**) SEM comparison of NET structure and thickness released by canonical neutrophils or PMN-DCs in response to *C*. *albicans* or *B*. *dermatitidis*. (**D**) *C*. *albicans* was incubated for 2 hours to become filamentous before neutrophils or PMN-DCs were added in killing assays in the presence or absence of 50 μg/ml DNase I. (**E**) WT mice were infected IV with 10^5^ *C*. *albicans* yeast and received 2 x 10^6^ canonical neutrophils or PMN-DCs (or PBS vehicle) IV 24 hours later. Burden in kidneys shown at 3 dpi. (A, D) *C*. *albicans* viability was determined by XTT assay and compared with neutrophil-absent control to calculate percent killing. (A) *B*. *dermatitidis* was plated on BHI agar after to determine number of remaining viable yeast. Means ± SEM are shown. Data are representative of at least two independent experiments.

In generating highly purified PMN-DCs from ER-HoxB8 GMPs, we addressed questions not testable *in vivo* or with mixed neutrophil populations. By incubating purified PMN-DCs with *C*. *albicans* or *B*. *dermatitidis*, we confirmed that PMN-DCs directly kill the fungi. This highly enriched population of PMN-DCs killed both *C*. *albicans* and *B*. *dermatitidis* significantly better than did undifferentiated neutrophils (Fig 6A). PMN-DCs killed ≈70% of *C*. *albicans* after a 4-hour incubation. Because PMN-DCs kill fungi directly, we analyzed interactions between fungi and PMN-DCs or canonical neutrophils by scanning electron microscopy. As we noted above (Fig 3A), PMN-DCs frequently phagocytosed *B*. *dermatitidis*, whereas canonical neutrophils did not successfully phagocytose this yeast (Fig 6B, S11 Fig). We also observed that PMN-DCs released NETs against *B*. *dermatitidis* and *C*. *albicans* (Fig 6B, S11 Fig). Interestingly, NETs released by PMN-DCs appeared to be thicker and associated with more protein-like material than NETs released by canonical neutrophils (Fig 6C). To further investigate the role of NETs in killing by PMN-DCs, we induced *C*. *albicans* to filament (S11 Fig) before adding canonical neutrophils or PMN-DCs. NETs are known to kill *Candida* [45,46], and filaments here induced greater NETosis. Although canonical neutrophils killed *C*. *albicans* better after it filamented, the PMN-DCs still killed *C*. *albicans* to a greater degree (>80%) than did canonical neutrophils (Fig 6A and 6D). To determine the contribution of NETs to killing, we added DNase to killing assays to degrade NETs. DNase significantly decreased killing by both canonical neutrophils and PMN-DCs, suggesting that NETs contribute to PMN-DC-mediated killing of *Candida* (Fig 6D).

### Adoptive transfer of PMN-DCs during systemic fungal infection

A major benefit of deriving PMN-DCs from ER-HoxB8 GMPs is that a large number of highly pure PMN-DCs are readily obtained without costly or detrimental enrichment procedures. Thus, adoptive transfer of pure PMN-DCs in numbers is feasible. Because PMN-DCs kill *C*. *albicans* well *in vitro*, we administered PMN-DCs (or canonical neutrophils) into wild-type mice with systemic candidiasis to test whether the cells augment antifungal immunity and have the potential as an adjunctive therapy. A single transfer of PMN-DCs, but not canonical neutrophils, significantly reduced the fungal burden in the kidneys of mice with systemic candidiasis (Fig 6E). Thus, PMN-DCs may be a good candidate for cellular immunotherapy against invasive fungal infections.

### Presentation of fungal antigen by PMN-DCs

One key function that distinguishes PMN-DCs from canonical neutrophils is the capacity to process and present antigen and prime T cells. Much of the work investigating antigen presentation has used mixed neutrophil populations (comprised largely of canonical neutrophils) in *in vitro* assays with T cells [3]. Investigation of *in vivo* presentation by PMN-DCs has relied on presentation of model OVA peptide to OT-II cells [5]. To expand understanding of antigen presentation by PMN-DCs and investigate fungal antigen presentation by PMN-DCs, we employed 1807 TCR transgenic CD4^+^ cells (Tg1807) that recognize fungal calnexin from multiple pathogenic fungal ascomycete species and confer vaccine immunity [28].

**Fig 7:**
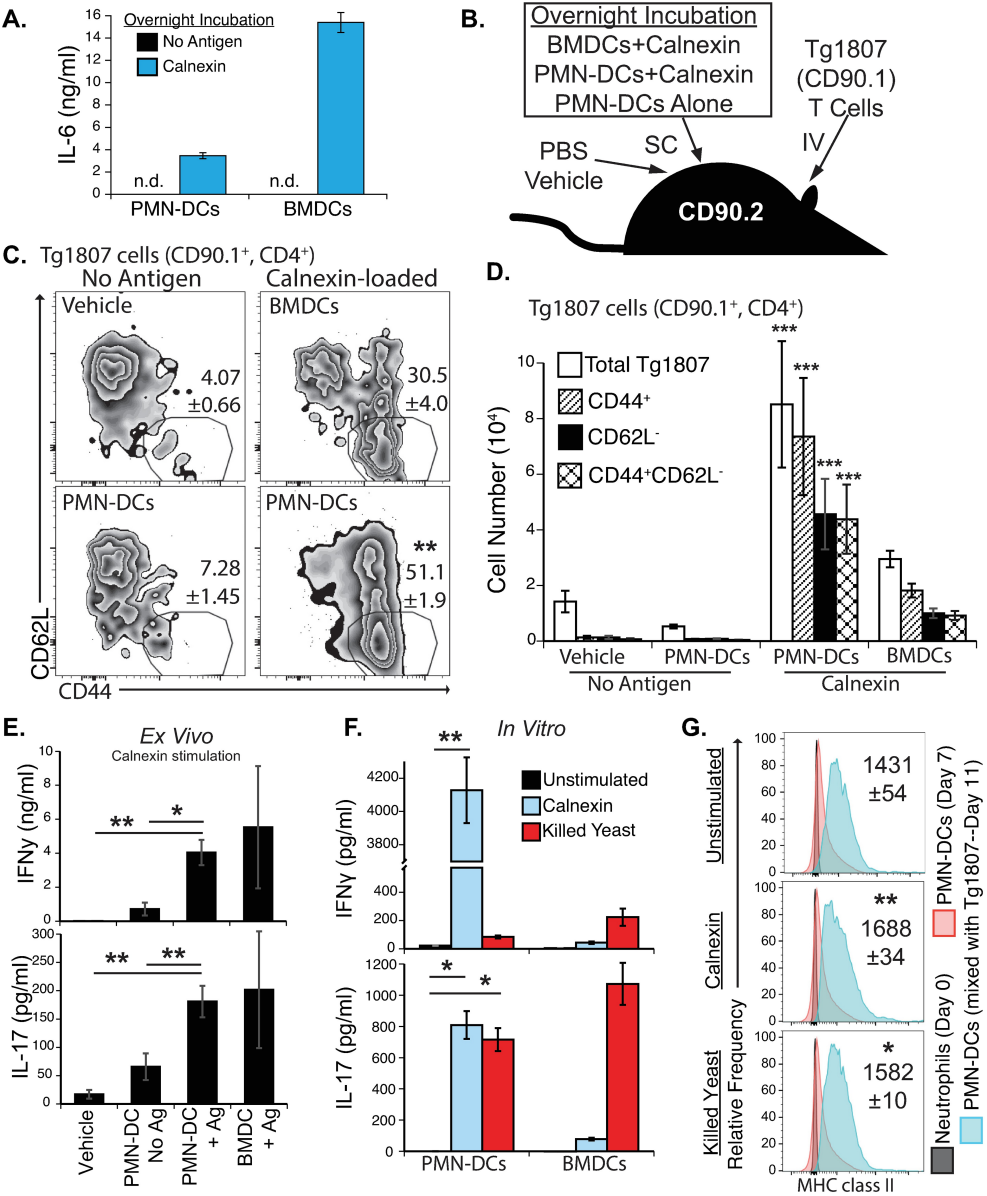
Presentation of fungal antigen (calnexin) to transgenic Tg1807 T cells by PMN-DCs generated *in vitro* from ER-HoxB8 GMP cells. (**A**–**D**) PMN-DCs or bone-marrow DCs (BMDCs) were incubated overnight with or without recombinant fungal calnexin, and the next day 5 x 10^4^ cells were injected subcutaneously into CD90.2 mice that had received adoptive transfer of Tg1807 cells (CD90.1^+^, calnexin-specific CD4^+^ T cells). One week later skin draining lymph nodes were harvested. **(A)** IL-6 in supernatants from overnight culture of PMN-DCs or BMDCs with or without calnexin, n.d,:not detected. **(B)** Experimental design of delivering calnexin-loaded APCs into mice that had received congenic Tg1807 cells. **(C)** Proportion of Tg1807 cells activated in lymph nodes (indicated by CD44^+^ and CD62L^-^). **(D)** The absolute number of Tg1807 cells and activated Tg1807 cells for each treatment group (N = 5–6 mice). (**E**) Lymph node cells (as shown in C-D) were stimulated *ex vivo* for 3 days with calnexin, and IFN-γ and IL-17 assayed in culture supernatants, Ag: antigen pulsed. (**F-G**) PMN-DCs or BMDCs were incubated overnight with fungal calnexin or heat-killed *Blastomyces* yeast before enriched CD4^+^ Tg1807 cells were added. (F) Presence of IFN-γ and IL-17 in supernatants collected after 3 days was determined by ELISA. (G) Expression of MHC class II from PMN-DCs at the end of the assay (Day 11) shown with comparison of MHC class II expression before mixing with T cells (Day 7) and before PMN-DC differentiation (Day 0), MFI indicated (±SEM). Both *in vivo* and *in vitro* experiments were completed independently twice. Statistics: for C-E, statistical significance between calnexin-loaded PMN-DCs and no antigen controls; for F-G, statistical significance between samples with fungal antigen and unstimulated.

To investigate the capacity of PMN-DCs to process and present fungal antigen, we generated PMN-DCs from GMP cells (as in Fig 5E) and incubated them with recombinant fungal calnexin. Fungal calnexin induced IL-6 production by PMN-DCs (Fig 7A). PBS vehicle control, unstimulated PMN-DCs, antigen-pulsed PMN-DCs, or antigen-pulsed bone marrow DCs (BMDCs; a positive control) were injected subcutaneously into mice that had received a transfer of naïve Tg1807 cells (Fig 7B). After 7 days, we analyzed the activation of Tg1807 cells in skin draining lymph nodes. Activated (CD44^+^CD62L^-^) Tg1807 cells were increased in mice that had received calnexin-loaded PMN-DCs, but not in mice that had received vehicle or control PMN-DCs (Fig 7C). Calnexin-pulsed PMN-DCs induced a 15-fold increase in the number of Tg1807 cells, and over a 100-fold increase in the number of activated Tg1807 cells in draining lymph nodes, when compared to recipients of control PMN-DCs (Fig 7D). Delivery of calnexin-loaded PMN-DCs induced an antigen-specific response because the response among endogenous CD4^+^ and CD8^+^ T cells was undetectable after delivery of PMN-DCs pulsed or not with calnexin (S12 Fig). Calnexin-pulsed PMN-DCs activated antigen specific T cells as well or better than calnexin-loaded BMDCs (Fig 7C and 7D, S13 Fig).

We also investigated the recall response of Tg1807 cells that were primed *in vivo*. Lymph node samples from the groups were incubated with recombinant calnexin *ex vivo* for 3 days to induce cytokine production in primed Tg1807 cells. We analyzed IFN-γ and IL-17, which are important cytokines of CD4+ T cells that promote antifungal immunity [15]. Upon *ex vivo* stimulation, cells from mice that had received antigen-pulsed PMN-DCs produced significantly more IFN-γ and IL-17 than cells from animals that received vehicle control (Fig 7E).

We considered a caveat in our *in vivo* assay. When antigen-pulsed PMN-DCs were delivered into mice, other antigen presenting cells could have received the calnexin to present to T cells, for example through *in vivo* antigen transfer. To establish that PMN-DCs present calnexin, we mixed PMN-DCs with freshly enriched CD4^+^ T cells from naïve Tg1807 mice in *in vitro* antigen-presenting assays. Calnexin-loaded PMN-DCs induced robust T cell production of IFN-γ and IL-17, and yeast-loaded PMN-DCs induced strong IL-17 production (Fig 7F, S14 Fig). PMN-DCs in the absence of T cells did not produce IFN-γ or IL-17 (S14 Fig).

Several studies have linked T cell production of cytokines with expression of MHC class II on neutrophils [3]. One study showed that human memory T cells induced MHC class II expression on neutrophils (with canonical phenotype) *in vitro* and that this induction was dependent on presence of cognate antigen [47]. In our *in vitro* antigen presentation experiment, we asked whether Tg1807 cells induce expression of MHC class II on PMN-DCs, particularly because *in vitro* derived PMN-DCs tend to have low MHC class II expression (Fig 5). We found that PMN-DCs cultured in the presence of T cells had an increase in MHC class II surface expression, and this expression was slightly enhanced by the presence of calnexin or killed *B*. *dermatitidis* (Fig 7G). In sum, PMN-DCs present fungal antigen and prime T cells. Antigen-pulsed PMN-DCs primed type 1 and type 17 responses known to be protective against fungal infections (S15 Fig).

## Discussion

Herein, we found that neutrophils trans-differentiate into PMN-DCs during fungal infections. PMN-DCs differentiate from canonical neutrophils under local and systemic inflammation in humans and mice regardless of the inducer of inflammation [3]. We did see greater differentiation of PMN-DCs during pulmonary fungal infection than what has previously been reported in inflamed murine lungs [5,48]; still, a minor proportion (<2%) of neutrophils became PMN-DCs. What is remarkable about this relatively small population of leukocytes is their outsized capacity to associate with and kill fungal cells. PMN-DCs comprised ≈1% of the total leukocytes during pulmonary blastomycosis but accounted for ≈15% of the yeast killed in the lungs. A defining characteristic of PMN-DCs is that they retain neutrophil antimicrobial functions even after evolving into antigen-presenting cells [4]; nevertheless, PMN-DCs associated with fungal cells and killed them at higher rates than canonical neutrophils.

PMN-DCs have been observed under various settings, including infections such as Leishmaniasis [11], autoimmunity in the joints of arthritis patients [8], tumors [12] and allergic response [5]. Despite their presence in these maladies, no clear functional role has been demonstrated for PMN-DCs in resolving or exacerbating disease. PMN-DCs might promote inflammatory bowel disease or anti-tumor immunity since PMN-DCs from the bowel or tumors can present antigens, including tumor antigens, *ex vivo* [9,12]. However, whether PMN-DCs play a direct role in damage or microbial killing has been overlooked because their population size is small compared to canonical neutrophils that possess these same functions. We used two powerful tools including DsRed fungal strains and a neutrophil progenitor line to compare fungal killing between canonical neutrophils and PMN-DCs. The DsRed fungal strains allowed us to identify leukocytes that kill fungi *in vivo* [35,42]. We generated high purity (>90%) PMN-DCs by culturing and differentiating a GMP cell line [44]. Our results with DsRed fungi and cell line-derived neutrophils indicate that PMN-DCs are highly effective killers of fungi and major contributors to antifungal immunity. We also demonstrated that adoptive transfer of PMN-DCs ameliorates disease by significantly reducing fungal burden.

### Mechanisms of enhanced killing

During pulmonary blastomycosis, PMN-DCs associated with yeast better than any other leukocyte. Yeast association rates were nearly 100-fold greater for PMN-DCs compared to canonical neutrophils. Association rates with *A*. *fumigatus* spores also were much higher for PMN-DCs than for canonical neutrophils both *in vivo* and *in vitro*. One explanation for this finding is the higher expression of surface PRRs on PMN-DCs than canonical neutrophils.

PMN-DCs retain the neutrophil feature of high phagocytic capacity [3]. Bone marrow-derived PMN-DCs phagocytose bacteria at the same rate as canonical neutrophils, but engulf latex beads at much higher rates [4]. Murine PMN-DCs that emerge during peritonitis phagocytose *E*. *coli* better than other peritoneal neutrophils or DCs [5]. Also, HLA-DR^+^ neutrophils enriched from the peripheral blood of patients phagocytose *Leishmania* promastigotes better than HLA-DR^-^ neutrophils [11]. We tracked phagocytosis *in vivo* and found that PMN-DCs phagocytosed many more yeasts than canonical neutrophils, accounting for ≈40% of the phagocytosed yeast in the lung. By using cell line-derived PMN-DCs, we confirmed that PMN-DCs phagocytosed yeast more efficiently than did canonical neutrophils. The increased size of PMN-DCs probably promotes enhanced phagocytosis, as particle size physically constrains phagocytosis [45]. At 8–10 μm, *B*. *dermatitidis* yeast are nearly as large as canonical neutrophils [49], so that larger PMN-DCs (~20 μm) are better able to phagocytose *B*. *dermatitidis* and other larger organisms such as *Leishmania* promastigotes.

ROS and NO are essential neutrophil products for killing fungi [1,17,40]. PMN-DCs are known to produce ROS, but to our knowledge, we report the first instance of NO production by PMN-DCs. We found that NO production was similar for PMN-DCs and canonical neutrophils, whereas ROS production was greater in PMN-DCs than canonical neutrophils. Two studies of human HLA-DR^+^ neutrophils noted enhanced ROS production after *in vitro* stimulation [11, 50]. We, too, saw that PMN-DCs from *Blastomyces*-infected lungs produced more ROS than canonical neutrophils when stimulated *ex vivo* with f-MLP.

NETs are a neutrophil defense against large organisms such as filamentous fungi [45]. *Candida* and other fungi pathogens are susceptible to killing by NETs [46]. PMN-DCs release NETs in response to PMA [4], and we show that fungi also induce NETosis by PMN-DCs. Because fungal killing was reduced in the presence of DNase, NETs from PMN-DCs appear to play a role in fungal killing. We also noted that NETs released by PMN-DCs appear thicker and have larger aggregates of protein-like material than NETs from canonical neutrophils; it is possible that NETs made by PMN-DCs could be laden with more antimicrobials and display greater killing potential.

In sum, in the context of fungal infection, PMN-DCs have similar or enhanced antimicrobial functions compared to canonical neutrophils. Increased surface PRR expression, phagocytosis, ROS production, and NETosis after differentiation into PMN-DCs likely explain how PMN-DCs associate with and kill fungal cells so effectively.

### PMN-DCs in immunity against aspergillosis and candidiasis

The most common, life-threatening fungal infections are caused by *Asperigillus* and *Candida* species [21]. We investigated the expansion of PMN-DCs in murine models of pulmonary aspergillosis and systemic candidiasis. Wild-type mice rapidly clear *Aspergillus* in the lung; however, wild-type mice quickly die with systemic candidiasis [43]. During pulmonary aspergillosis, we saw robust expansion of neutrophils that expressed both CD11c and MHC class II and killed spores with greater efficiency than did canonical neutrophils and moDCs. During systemic candidiasis, neutrophil differentiation was incomplete with few CD11c^+^MHCII^+^ appearing particularly in the primary target organ, the kidney. Because *in vitro* differentiated PMN-DCs kill *C*. *albicans* efficiently *in vitro*, the limited numbers and differentiation of PMN-DCs during systemic candidiasis may contribute to the eventual mortality. In this regard, it is noteworthy that adoptive transfer of fully differentiated PMN-DCs significantly decreased *C*. *albicans* in the kidneys. PMN-DCs may offer cell therapy strategies to be studied for improving outcomes during systemic candidiasis or pulmonary aspergillosis.

### Generation of PMN-DCs from a neutrophil progenitor cell line

Investigations of PMN-DCs have been greatly limited by the difficulty in obtaining a large source of pure PMN-DCs. Studies investigating PMN-DCs in murine models have either used mixed populations of neutrophils or relied on FACS sorting [4,5,9]. Work with murine PMN-DCs is also complicated by the fact that primary neutrophils require feeder cells to differentiate *in vitro* [4,6]. To circumvent these issues, we employed a GMP cell line to produce neutrophils that we then differentiated into PMN-DCs [44]. This cell line was a continual, consistent source of PMN-DCs that did not rely on an animal source. Additionally, we successfully generated PMN-DCs without the use of feeder cells, greatly simplifying their production.

PMN-DCs generated from the GMP cell line resembled PMN-DCs differentiated from bone marrow neutrophils [4]: they displayed high amounts of CD11c, an increase in surface MHC class II, and morphological changes consistent with differentiation into a DC-like cell. The cell line-derived PMN-DCs were also functionally similar to *in vivo* PMN-DCs e.g. they associated with and killed fungal cells better than canonical neutrophils. Further, generating PMN-DCs from a cell line increases the feasibility of experiments probing the biology of PMN-DCs. For example, we verified that PMN-DCs directly kill fungal cells and produced NETs in response to fungi. We also used GMP-derived PMN-DCs to investigate presentation of fungal antigen and polarization of T cell responses. Finally, by using the cell line, we obtained enough pure PMN-DCs to show that adoptive transfer of PMN-DCs conferred immunity to systemic candidiasis.

### Presentation of fungal antigen

While human neutrophils have been shown to present disease-associated antigens to T cells *in vitro*, studies of murine neutrophils have been limited to presentation of OVA peptide to OT-I and OT-II cells [3,12,47]. We used Tg1807 CD4^+^ T cells, which recognize the pan-fungal antigen calnexin and confer protective immunity against various fungal infections [28]. Delivery of calnexin-pulsed PMN-DCs activated Tg1807 cells *in vivo* and promoted Th1 and Th17 recall responses. PMN-DCs given either recombinant calnexin or heat-killed *B*. *dermatitidis* also presented antigen to Tg1807 cells *in vitro* inducing production of IFN-γ and IL-17. While this confirms prior studies that murine neutrophils polarize Th1 and Th17 *in vitro* [5,9,50], type 1 and type 17 responses are essential for protective immunity against fungal infections including candidiasis, aspergillosis and blastomycosis [15]. By showing that antigen-pulsed PMN-DCs induce Th1 and Th17 in antigen-specific T cells, we unveil a role for PMN-DCs in arming adaptive immunity concomitantly while directly killing fungi (S15 Fig).

T cells, by producing cytokines, are implicated in inducing neutrophil differentiation into PMN-DCs. Human mucosal innate T cells induce PMN-DC differentiation *in vitro* and this differentiation is inhibited by neutralizing antibodies against T cell-produced cytokines [51]. Human memory T cells induce MHC class II expression on autologous neutrophils *in vitro*, but only in the presence of cognate antigen [47]. Murine T cells in the absence of antigen induce MHC class II expression on neutrophils *in vitro* [52]. We too looked at MHC class II expression in PMN-DCs differentiated from the GMP cell line. Like PMN-DCs generated from bone marrow neutrophils, GMP-derived PMN-DCs have low expression of MHC class II^+^ after differentiation. MHC class II expression increased significantly in the presence of T cells, although expression was only slightly increased after concomitant incubation with cognate antigen. We also noted increased MHC class II expression in GMP-derived PMN-DCs incubated overnight with *A*. *fumigatus* spores, but for this assay feeder cells were present, indicating that signals from these cells may influence MHC class II expression on neutrophils.

### Implications of PMN-DCs in antifungal therapy

Invasive fungal infections are a leading cause of death in cancer and transplant patients [20–25], and in patients requiring intensive care [18]. Treatment of deadly fungal infections relies heavily on antifungal drugs, but antifungals have limitations in bioavailability, drug resistance and toxicity [26]. Because antifungals cannot fully control some fungal infections, immunotherapeutic approaches are being sought [31]. Neutrophils are essential for immunity to most fungal diseases, including aspergillosis and candidiasis [16]. Impaired neutrophil immunity is a major risk factor for invasive fungal infection and mortality, highlighting that neutrophils are crucial targets of immunotherapy.

Our current work supports the targeting of this unique population of neutrophils, PMN-DCs, in treating invasive fungal infections. PMN-DCs are better cellular targets than therapies directed at canonical neutrophils because (1) PMN-DCs better engage and kill fungi; (2) PMN-DCs persist longer than canonical neutrophils, limiting the number or duration of treatments needed and saving cost and patient stress; (3) PMN-DCs arm adaptive immunity, promoting better defense and long-term protection, thereby reducing the need for continual intervention.

Harnessing PMN-DCs to fight lethal fungal infections may only require modifications of therapies in practice that enhance neutrophil immunity. Adjunctive cytokine therapy increases circulating neutrophils in neutropenic patients and has helped in treating fungal infections [31]. Additionally, granulocyte transfusion, out of practice for some time, is now effective with appropriate donor neutrophil preparation [53]. Cytokine therapies could be modified to promote expansion of PMN-DCs in patients, or PMN-DCs could be differentiated from autologous or donor neutrophils *ex vivo* prior to transfusion. Although work is needed to determine how amplified PMN-DCs would affect patients, we show that these cells arise naturally during infection, and they likely increase in certain widely practiced immunotherapies. We also show that adoptive transfer of PMN-DCs confers protection during systemic infection. In patients at greatest risk from fungal infections, harnessing PMN-DCs could improve disease outcomes.

## Materials and methods

### Mice

Wild-type C57BL/6 were purchased from Charles River Laboratories (Wilmington, MA). Mice challenged with fungi or recipients of Tg1807 cells were 7–13 weeks old. Bone marrow used to culture PMN-DCs or BMDCs *in vitro* came from mice up to 16 weeks old. C57BL/6 Tg1807 CD90.1 mice [28] were bred in house; spleens and lymph nodes were harvested from Tg1807 mice between 8–18 weeks old. C57BL/6 *ccr2*^-/-^ and ubiquitin C (UBC)-GFP mice were purchased from Jackson Laboratory (Bar Harbor, ME). All procedures with mice were in accordance with a protocol approved by the University of Wisconsin Animal Care and Use Committee, and in line with accreditation by the American Association for Accreditation of Laboratory Animal Care and NIH guidelines.

### Fungi

*Blastomyces dermatitidis* wild-type strain ATCC 26199 or engineered to express DsRed or GFP [35] were used in this study and maintained by passage on 7H10 slants. Anesthetized mice were challenged IT with 2 x10^4^ yeast suspended in 20 μl of PBS unless otherwise noted. CFUs were quantified after experiments by spreading yeast on brain-heart infusion (BHI) agar plates after 6–7 days at 37°C. In some experiments, yeast were stained with 20 μg/ml Uvitex 2B (PolySciences, Inc., Warrington, PA), a fluorescent chitin stain. Yeast were heat-killed by incubation at 70° C for 40–60 minutes.

*Aspergillus fumigatus* strain Af293 expressing DsRed, a kind gift from Tobias Hohl [42], was grown on glucose minimal medium plates for 7 days at 37°C. Spores were harvested from plates with 0.01% TWEEN-80 water and strained to remove hyphae. Spores were stained with 10 μg/ml Uvitex for *in vitro* experiments or 40 μg/ml Uvitex before IT challenge. Before some *in vitro* experiments, spores were biotinylated and stained with streptavidin-Alexafluor633 (Af633) [40,42]. For infection, mice were challenged with 4 x10^7^ spores IT.

*Candida albicans* strain SC5314 was grown from frozen stocks on yeast peptone dextrose (YPD) agar plates for 1–2 days at 30°C, and colonies were picked and grown in YPD medium overnight in a 30°C shaking incubator. After overnight culture, yeasts were prepared for challenge or *in vitro* assays. Anesthetized mice were challenged IV with 1.0–2.5 x 10^5^ yeast in 0.5 mL PBS via the retro-orbital vein [43].

### Tissue collection and processing

Lungs were harvested, processed, and digested with collagenase D and DNase I (Sigma, St. Louis, MO), as described [35]. For experiments tracking fungal cells, to improve staining, mice were bled and hearts were perfused with 5–10 mL of PBS after the animals were euthanized. In some experiments where neutrophil populations were tracked, mice were injected IV with 2 μg/ml anti-CD45 (30-F11) (Biolegend, San Diego, CA) to stain leukocytes in the capillaries prior to euthanizing and harvesting lungs [33,54]. Lungs were harvested 2 days post infection (dpi) from mice challenged with *A*. *fumigatus* spores and between 1–14 dpi from mice challenged with *B*. *dermatitidis*.

Peripheral blood, spleens and kidneys were collected from naïve mice or mice 1–3 dpi with *C*. *albicans*. Peripheral blood was collected from the retro-orbital vein into heparin and mixed with an equal volume of 2% dextran (450–650 kDa, Sigma) to aggregate erythrocytes for 45–60 minutes at room temperature. The volume suspended over erythrocyte aggregates was collected and cells were washed before lysing the remaining erythrocytes with ACK (ammonium-choride-potassium) buffer [35].

Kidneys were minced, then crushed with a syringe plunger. Following mechanical disruption, kidneys were digested with 1 μg/ml collagenase I, 1 μg/ml collagenase II, 1 μg/ml collagenase IV (Worthington Biochemical, Lakewood, NJ) and 20 μg/ml DNase I (Sigma) in RPMI-1640 without inactivated fetal bovine serum (FBS) for 30 minutes at 37°C. After digest and erythrocyte lysis, cells were resuspended in 40% Percoll:60% RPMI (with 1% FBS). Cells in 40% Percoll were underlaid with 80% Percoll and centrifuged for 20 minutes at 800 x *g* at room temperature [43]. The interface was collected, and cells were stained for flow cytometry.

Spleens were dissociated with a syringe plunger through 40–70 μm mesh and splenic erythrocytes lysed in ACK buffer. Lymph nodes were also dissociated with a syringe plunger through 40–70 μm mesh. For collection of lymph nodes from Tg1807 mice, inguinal, axillary, brachial, and cervical lymph nodes were collected and pooled. For mice that received subcutaneous (SC) injections, skin-draining inguinal, axillary and brachial lymph nodes were collected and pooled.

### Flow cytometry

Leukocytes were stained in PBS containing 0.5% bovine serum albumin. For all experiments cells were incubated with Fc Block (anti-mouse CD16/CD32) (BD Biosciences, San Jose, CA) to limit surface Fc receptors binding to staining antibodies. Staining cocktails contained fluorescent-conjugated antibodies from BioLegend, BD Biosciences, or eBiosciences (ThermoFisher, Waltham, MA) unless noted otherwise. Antibodies were directed against the following markers (clones noted in parenthesis): CD4 (RM4-5 or GK1.5), CD8a (53–6.7), CD11a (M17/4), CD11b (M1/70), CD11c (N418 or HL3), CD40 (3/23), CD44 (IM-7), CD45/Ly5 (30-F11), B220/CD45RA (RA3-6B2), CD62L (MEL-14), CD64 (X54-5/7.1), CD69 (H1.2F3), CD80 (16-10A1), CD86 (GL-1), CD90.1/Th1.1 (OX-7), CD90.2/Th1.2 (53–2.1), CD103 (M290), CXCR2/CD182 (SA045E1), Mannose Receptor/CD206 (C068C2), Langerin/CD207 (RMUL.2), TLR-2/CD282 (T2.5), TLR-4/CD284 (SA15-21), Dectin-1 (RH1), Galectin-3/Mac-2 (M3/38), MHC class I H-2Kb/H-2Db (28-8-6), MHC class II I-A/I-E (M5/114.15.2) Ly6C (HK1.4 or AL-21), Ly6G (1A8), F4/80 (BM8), NK1.1 (PK136), Siglec F (E50-2440), Siglec H (551).

Unconjugated rat anti-murine Dectin-2 (D2.11E4) (ThermoFisher, Waltham, MA) and anti-murine Mincle (4A9) (MBL, Woburn, MA) were used as primary antibodies incubated alone with cells. After washing, PE or APC conjugated anti-rat IgG2a (BD Biosciences) was included in a cocktail containing the other fluorescent conjugated antibodies. FMO controls for Dectin-2 and Mincle did not receive primary antibody but were stained with anti-rat secondary antibody.

To mark *B*. *dermatitidis* yeast *ex vivo* with Uvitex [35,55], after lung processing, cells were stained for surface markers, washed, and fixed with BD cytofix/cytoperm. Cells were then stained with 1 μg/ml Uvitex 2B in BD perm/wash buffer for 30 minutes at room temperature before being washed with perm/wash buffer. Intracellular staining of fungi with Uvitex also stains phagocytic cells in the lungs of naïve mice; these stained cells have likely engulfed chitin or other polymers inhaled from food or bedding [56]. These cells are present in small number in the lungs of mice infected with *B*. *dermatitidis*; they have dim Uvitex staining and also are autofluorescent in many channels including DsRed. These false positive events were gated out from the cells determined to be Uvitex^+^ as shown by Wang et al [55].

To track phagocytosis of *B*. *dermatitidis* yeast *in vivo*, cells from processed lungs were surface stained with 1 μM calcofluor white M2R (Sigma) in stain cocktails for 20 minutes at 4° C.

For all experiments, cells were stained with Invitrogen Live/Dead Fixable Yellow or Near Infared (ThermoFisher) to gate out dead cells. Forward and side scatter gates were also used to remove debris and, in experiments not investigating fungal cell association, to remove doublets (FSC-A X FSC-H, SSC-A x SSC-H).

Flow cytometry was performed on either a 3 or 5 laser BD LSRII or 5 laser BD Fortessa cytometers. Imaging cytometry was performed using an ImageStream MarkII (Amnis). FACS sorting was completed on a BD FACS Aria II. FACS was performed on live cells cultured from bone marrow or on fixed cells from lungs infected *B*. *dermatitidis*. Most flow cytometry was performed at the University of Wisconsin Carbone Cancer Center Flow Cytometry Core. Flow cytometry data was analyzed and plots were designed using Flowjo10 (Tree Star, Ashland, OR). Mean fluorescence intensities (MFI) were calculated in Flowjo using the geometric mean of fluorescence. Relative expression of surface proteins on cells was determined by subtracting the MFI of that marker’s fluorescence minus one (FMO) control on the population from the stained sample MFI of the population.

### Microscopy

Microscopy was completed with an Olympus BX60 microscope. Images were captured using QCapture Pro 6.0 and an Exi Aqua Camera (QImaging, Surry, BC) at room temperature. All images were captured under 40X or 100X power magnification. Raw images were cropped and resolution enhanced in Microsoft PowerPoint.

To ascertain the morphology of PMN-DCs, lungs were harvested 7 dpi from mice challenged with *B*. *dermatitidis* as described above. Cells were fixed with 2% paraformaldehyde and then FACS sorted to obtain PMN-DCs (CD11b^+^, Ly6G^+^, Ly6C^int^, CD11c^+^), canonical neutrophils (CD11b^+^, Ly6G^+^, Ly6C^int^, CD11c^-^), or moDCs (CD11b^+^, Ly6G^-^, Ly6C^+^, CD11c^+^). After sorting, cells were centrifuged onto a slide using a cytospin and stained with a Hema3 kit (ThermoFisher, Waltham, MA).

Morphology of ER-HoxB8 GMP derived cells (GFP^+^) was tracked after maturation of cells to neutrophils. Sterilized coverslips were placed in dishes before the addition of neutrophils and differentiation medium. Coverslips were moved to slides through the course of differentiation to capture images of cells. GMP cells through the course of differentiation also were centrifuged on to a slide and stained with a Hema3 kit.

To track interactions between fungal cells and neutrophils *in vitro*, canonical neutrophils or PMN-DCs, derived from GMP cells, were incubated for 3 hours at 37°C with *C*. *albicans* or *B*. *dermatitidis* yeast (prestained with 20 μg/ml Uvitex) in Lab-Tek chamber slides (Thermo Fisher). Before visualizing cells, propidium iodide (Sigma) was added to cultures at a final concentration of 40 μM and incubated at 37° C for 15 minutes. To wells with *C*. *albicans*, Uvitex was also added at a final concentration of 20 μg/ml with propidium iodide.

### *Ex vivo* staining of ROS and NO

After processing lungs, a subset of cells was incubated with either ROS or NO fluorescent indicators dihydrorhodamine-123 (DHR-123) (Chemodex [Adipogen, San Diego, CA]) or DAF-FM diacetate (4-amino-5-methylamino-2’,7’-difluorofluorescein diacetate) (Cayman, Ann Arbor, MI) at 37° C [39]. To quantify ROS production, cells were incubated with 10 μg/ml DHR-123 in RPMI with 10% FBS for 3 hours if unstimulated or 30 minutes with 10nM N-formyl-L-methionyl-L-leucyl-phenylanalynine (f-MLP) [33]. To quantify NO production, unstimulated cells or cells pre-incubated 60 minutes with 2 μg/ml lipopolysaccharide (LPS) were incubated with 100 nM DAF-FM diacetate in PBS for 10 minutes. After incubation with indicator dyes, cells were stained for surface markers as described above.

### Generating PMN-DCs from bone marrow

The marrow of wild-type mice was harvested from femurs and tibias as previously described [35]. Cells were resuspended in RPMI with 10% FBS and 10 ng/ml GM-CSF and 1 ng/ml IL-4. Cells were cultured in 6-well plates at a density of 4–6 x 10^6^ cells/well. Medium was refreshed every other day. Non-adherent cells were collected at day 6 and co-cultured with uvitex-stained fungal cells overnight in RMPI with 10% FBS at 37°C, then prepared for flow cytometry. Day 6 cells were also FACS sorted to purify canonical neutrophils and PMN-DCs. These sorted cells were resuspended in RMPI with 10% FBS and mixed with *B*. *dermatitidis* yeast at an effector-target ratio of 3:1 and incubated overnight at 37°C; then cells were lysed, and yeasts were plated on BHI agar to enumerate CFU.

### Generating PMN-DCs from a neutrophil progenitor cell line

Bone marrow mononuclear cells isolated from a UBC-GFP mouse (Jackson strain 004353) were transduced with retrovirus (MSCVneo) expressing the ER-HoxB8 (estrogen receptor) fusion protein as described by Wang et al [44]. Single-cell GMP clones were selected that uniformly matured into neutrophils as assessed by cell surface staining, function, and morphology. GMPs were maintained as progenitors in estrogen (beta-estradiol, 0.5 μM) and conditioned medium containing stem cell factor (~100 ng/ml) and matured into neutrophils for 4–5 days in conditioned medium lacking estrogen. After cells were matured into neutrophils, they were centrifuged at low speed (150 x *g*) to remove dead cells and cultured for 5–7 days under conditions that promote PMN-DC differentiation. Neutrophils were differentiated in RPMI with 10% FBS and 10 ng/ml GM-CSF and 1 ng/ml IL-4 [6]. For initial experiments, neutrophils were cultured with bone marrow feeder cells, prepared as described above, at a ratio of 3 feeder cells to 1 neutrophil; later experiments did not use feeder cells. GFP-expression was used to distinguish the PMN-DC from feeder cells.

To assess functions of GMP-derived PMN-DCs, neutrophils before differentiation or PMN-DCs after differentiation were incubated with fungal cells or β-1,3-glucan coated beads. To track killing of *Aspergillus*, DsRed *A*. *fumigatus* spores, pre-stained with Uvitex, were co-cultured with neutrophils or PMN-DCs at an effector-to-target ratio of 3:1 for 12 hours, then stained for flow cytometry. To track association with fungal-like particles, AlexaFluor647 beads coated with the fungal cell wall component β-1, 3-glucan [57] were incubated with neutrophils or PMN-DCs for 1 hour at 37°C and then assessed by flow cytometry. To track killing of *B*. *dermatitidis*, yeasts were co-cultured with neutrophils or PMN-DCs overnight at an effector-to-target ratio of 3:1; cells were then lysed and yeasts were spread on BHI plates to quantify viability. To track killing of *C*. *albicans*, yeasts were co-cultured with neutrophils or PMN-DCs, or *C*. *albicans* was incubated for 2 hours to promote filamentation before addition of neutrophils or PMN-DCs. Co-cultures of *C*. *albicans* and cells were incubated for 4 hours at 37°C; neutrophils were then lysed with water, and yeast viability was quantified by XTT, 2,3-Bis(2-methoxy-4-nitro-5-sulfophenyl)-2*H*-tetrazolium-5-carboxanilide (ThermoFisher), assay, as previously described [58]. To investigate the role of NETs, 50 μg/ml DNase I (Sigma) was added to co-cultures of neutrophils or PMN-DCs with *C*. *albicans* hyphae at the beginning of the 4-hour co-culture.

### Scanning electron microscopy

Coverslips used for scanning electron microscopy (SEM) were prepared, as previously described [59–61]. Briefly, planktonic *C*. *albicans* or *B*. *dermatitidis* yeast were added to poly-L-lysine-treated plastic 13 mm diameter coverslips (Thermonax, ThermoFisher) and incubated for 1 hour at 30° C for *C*. *albicans* or 37° C for *B*. *dermatitidis*. Canonical neutrophils or PMN-DCs differentiated from the GMP cell line were added to wells containing coverslips to reach an effector-to-target ratio between 1:2 and 2:1, and co-cultures were incubated for 4 hours at 37° C. After washing, coverslips were fixed overnight in 4% formaldehyde and 1% glutaraldehyde, followed by washing and treatment with 1% osmium tetroxide. Samples were then washed, dehydrated, dried, and coated with 14 nm platinum. Microscopy was completed on a LEO 1530 scanning electron microscope at 3 kV.

### Adoptive transfer of PMN-DCs

Mice were challenged with 10^5^ *C*. *albicans* IV, as described above, and 24 hours later 2 x 10^6^ neutrophils or PMN-DCs, differentiated from ER-HoxB8 GMP cells as described above, were administered IV in 500 μl of PBS. For vehicle control, mice received 500 μl of PBS alone. At 3 dpi, kidneys were harvested and homogenized; CFU was quantified by plating kidney homogenates on YPD agar plates. Homogenates from both kidneys were combined and CFU/kidney was calculated by dividing CFU by 2.

### Assessment of antigen presentation

PMN-DCs were generated from the GMP cell line, as described above, by culturing neutrophils for 7 days with GM-CSF and IL-4. BMDCs were generated from bone marrow leukocytes with 20ng/ml GM-CSF for 7–10 days; for the first 2 days, the medium (RPMI+10%FBS) contained 5 ng/ml IL-4. BMDCs were harvested by collecting non-adherent cells.

For *in vivo* experiments, PMN-DCs or BMDCs were cultured alone or with recombinant calnexin (from P*aracoccidioides brasiliensis* [28]) at 50 ng/mL overnight. Supernatants were collected and cells were washed with PBS. PMN-DCs were resuspended to a concentration of 5 x 10^4^ cells/mL in PBS; 1 mL of cells or vehicle control were then injected SC into mice. Before mice (CD90.2) received calnexin-loaded cells or controls, they received an IV transfer of 2 x 10^6^ pooled cells from spleens and kidneys of CD90.1 Tg1087 mice. We determined that about 10% of the transferred cells from Tg1807 mice were CD4^+^ T cells, which have calnexin-specific TCRs, so all mice received approximately 2 x10^5^ Tg1807 cells IV. Seven days after antigen presenting cells were injected SC, skin-draining lymph nodes were collected and processed as described above before staining for flow cytometry. To investigate the recall responses of *in vivo* primed T cells, a subset of lymph node samples was cultured in RPMI with 10% FBS with 50 μg/ml calnexin for 3 days, and supernatant was collected for ELISA.

For *in vitro* antigen presentation, PMN-DCs or BMDCs were generated as described above and cultured overnight alone or with 100 μg/ml recombinant calnexin or heat-killed *B*. *dermatitidis* yeast at a ratio of 1:1. The next day CD4^+^ T cells from pooled spleens and lymph nodes of Tg1807 mice were positively enriched using anti-mouse CD4 magnetic particles-DM (BD Biosciences). Enriched CD4^+^ Tg1807 cells were added in equal volume to PMN-DCs or BMDCs, which had been incubated with or without antigen, at a ratio of 5:1 T cells:APCs. As a control, Tg 1807 cells were added at equivalent numbers to wells without any APCs, containing medium alone, calnexin, or heat-killed *B*. *dermatitidis*. Tg1807 cells were incubated with or without APCs for 3 days and supernatants were collected for ELISA. Remaining cells were stained to track PMN-DC expression of MHC class II by flow cytometry.

ELISA kits for murine IL-6, IL-17 and IFN-γ (R&D Systems, Minneapolis, MN) were used to quantify cytokines in cell supernatants.

### Statistical analyses

Statistical analyses were performed using Graphpad Prism 5. All experiments comparing populations of cells used statistical tests such as ANOVA with repeated measures, which grouped data coming from individual mice (for *in vivo* experiments) or cell culture wells (for *in vitro* experiments), ANOVA with Tukey post-hoc test was used to compare groups in experiments, unless population distributions were not normal in which case the non-parametric Kruskal-Wallace test was used. If only two groups were compared, a two-tailed Student’s *t*-test or Mann-Whitney test were used. Some data was log-transformed to permit use of parametric tests, especially when means differed on a log-scale. P-values displayed in figures are *p<0.05, **p<0.01, ***p<0.001.

## Supporting information

S1 Fig Phenotype of neutrophils and moDCs in naïve and *Blastomyces*-infected mice. (**A**) Neutrophils in the lungs of naïve mice are primarily constrained to the capillaries as indicated by staining by anti-CD45 that was injected IV just before euthanasia. These neutrophils are entirely CD11c^-^ with little or no MHC class II expression. Flow plots from a representative mouse shown on left and mean absolute number of neutrophils (±SEM) on right (N = 3). (**B**) Mice were challenged IT with 4 x 10^4^ *B*. *dermatitidis* and lungs were harvested 4 days later and prepared for flow cytometry. The lung leukocytes were analyzed by imaging cytometry. Representative images show a canonical neutrophil, a PMN-DC and a moDC. (**C**) Gating strategy for moDCs in the lung throughout the manuscript. The parent of the first plot is live (negative for live/dead stain) and CD11b^+^ Siglec F^-^. (**D**) Forward (FSC) and side (SSC) scatter area of canonical neutrophils, PMN-DCs and moDCs from lungs 7 days after challenge with *B*. *dermatitidis*. These data indicate that PMN-DCs are larger and more morphologically complex than canonical neutrophils and are morphologically more like moDCs.
(EPS)

S2 Fig Expression of surface markers on neutrophil populations and moDCs after infection with *B*. *dermatitidis*. (**A**) Expression of MHC class II on leukocyte populations. (**B**) Expression of CD40 showing histograms and mean MFI (minus FMO control fluorescence). (**C**) Expression of NK1.1 (natural killer cell marker), F4/80 (macrophage marker), CD11a (LFA-1 integrin), CD62L (L-selectin), and CD69 (a marker of activation). Representative histograms are shown. For panels B and C, red histograms are the stained populations and gray histograms are the FMO control for the same populations.
(EPS)

S3 Fig PMN-DCs do not appear to emerge from a lineage of inflammatory (Ly6C^hi^) monocytes. (**A**) Surface expression (MFI ± SEM indicated on representative histogram) of CD115 (M-CSF receptor, expressed by monocytes) on PMN-DCs in WT mice 7 dpi with *B*. *dermatitidis*; top shows the isotype control and the bottom shows the same sample stained with anti-CD115 antibody. (**B**) WT or *ccr2*^-/-^ mice (with deficiency in inflammatory monocyte recruitment [35]) were infected with *B*. *dermatitidis* and lungs were harvested 4 dpi; absolute number (±SEM) of PMN-DCs (Ly6G^+^, Ly6C^+^, CD11b^+^, CD11c^+^), inflammatory monocytes (Ly6G^-^, Ly6C^hi^, CD11b^+^, CD11c^-^), or moDCs (Ly6G^-^, Ly6C^+^, CD11b^+^, CD11c^+^). Data are representative 2 independent experiments (A) or 4 independent experiments (B); n = 5 mice for all groups; statistical comparisons were between populations of cells in WT or *ccr2*^-/-^ mice with a Mann-Whitney test, n.s.: not significant.
(EPS)

S4 Fig Surface expression of DC markers on neutrophil populations and moDCs from lungs of mice challenged with *B*. *dermatitidis* 7 days prior. (**A**) Surface expression of CD8a (alpha subunit), langerin (CD207 found on Langerhans cells) and CD103 (an integrin on resident DCs). (**B**) Expression of CD64 (high affinity monomeric IgG receptor characteristically expressed on murine moDCs) showing histograms and mean expression (±SEM) of indicated populations. (**C**) Expression of B220 (CD45R, found on pDCs) on neutrophils showing representative histograms (left panels; numbers denote % B220^+^) and mean expression (±SEM) of indicated populations (right panel). (**D**) B220 expression on neutrophils and expression of CD11c and Siglec H on B220^+^ MHC class II^+^ neutrophils with a comparison to plasmacytoid DCs (pDCs, bottom left) also from infected lungs. Representative histograms are shown in upper panels (N = 5 mice). Mean expression (shown in lower panel) was determined by subtracting fluorescence of FMO population from population MFI. Gray indicates FMO controls.
(EPS)

S5 Fig Association and killing of *B*. *dermatitidis* by PMN-DCs *in vivo* and *in vitro*. (**A**–**D**) DsRed *B*. *dermatitidis* yeasts were stained with Uvitex before IT challenge and lungs were harvested 48 hours later when Uvitex can still be detected. **(A)** Representative plots showing association of live (DsRed^+^) and killed (DsRed^-^) yeast (Uvitex^+^). **(B)** Percent killed denotes the proportion of yeast that are DsRed^-^ among the Uvitex^+^ yeast associated with indicated populations. **(C-D)** Percentage of the total live (C) or dead (D) yeast in the lungs associated with indicated populations. (**E**) Canonical neutrophils, PMN-DCs and moDCs were sorted from cultured bone marrow neutrophils (cultured with GM-CSF and IL-4 for 6 days) and incubated with *B*. *dermatitidis* yeast at an effector-to-target ratio of 3:1 overnight. After incubation, the yeast were plated for CFU and the killing rate was determined by calculating decrease in CFU compared to a control group that cultured yeast without leukocytes.
(EPS)

S6 Fig Surface expression of PRRs mannose receptor (CD206) and TLR-4 (CD284) on canonical neutrophils, PMN-DCs and moDCs in lungs 7 days after infection with *B*. *dermatitidis*. (**A**) Representative histograms and (**B**) mean expression (MFI above FMO control) of indicated populations. N = 5 mice. Red histograms are stained populations; gray histograms are FMO control populations.
(EPS)

S7 Fig *Ex vivo* stimulation of ROS and NO on PMN-DCs, canonical neutrophils and moDCs from lungs harvested 7 days after challenge with *B*. *dermatitidis*. (**A**) Leukocytes were stimulated *ex vivo* with f-MLP for 30 minutes in the presence of DHR-123 then stained for surface markers. (**B**) Leukocytes were stimulated *ex vivo* with LPS for 30 minutes in the presence of DAF-FM then stained for surface markers. Top rows show representative histograms (gray indicate unstained control) and bottom rows show the MFI of fluorescent probe and the percent positive populations. Means± is shown; N = 5 mice.
(EPS)

S8 Fig *In vitro* killing of DsRed *A*. *fumigatus* spores by cultured bone marrow leukocytes. Bone marrow leukocytes were cultured for 7 days with GM-CSF and IL-4 and incubated with spores at a 1:4 effector-to-target ratio for 6 hours and analyzed by flow cytometry. DsRed *A*. *fumigatus* spores are marked with Alexafluor 633 (Af633). (**A**) Concatenated plots showing association of live (DsRed^+^) and dead (DsRed^-^) spores (Af633^+^) with PMN-DCs, canonical neutrophils or moDCs. (**B**) Mean association (±SEM) rates of population with live and dead spores. (**C**) Killing rate (% DsRed^-^ of Af633^+^) for leukocyte populations.
(EPS)

S9 Fig Tracking differentiation of ER-HoxB8 GMP cells from GMPs to neutrophils. (**A**) ER-HoxB8 GMP are maintained in progenitor status in the presence of estrogen by promoting nuclear localization of HoxB8; once estrogen is washed from the medium HoxB8 no longer translocates to the nucleus promoting differentiation [44]. (**B-C**) Hema3 staining of GMP cells before differentiation (B) and after 4 days of differentiation (C) in the absence of estrogen and presence of stem cell factor (SCF); arrows indicate dividing cells, N: mature neutrophil, B: immature “band” neutrophil, Mm: metamyelocyte, My: myelocyte (metamyelocytes and myleocytes are neutrophil precursors [62]). (**D**) Expression of CD115 (M-CSF receptor, a monocyte marker) and F4/80 (a macrophage marker) on neutrophils differentiated from GMP cells after 4 days. (**E**) Like primary neutrophils, GMPs differentiated into neutrophils for 4 days were CD11b^+^ and Ly6C^+^, however GMPs lacked Ly6G expression that characterizes murine neutrophils. (**F-H**) Because the cell culture dish lacks signals that may allow for complete neutrophil maturation, we tracked neutrophil morphology and expression of Ly6G 24 hours after neutrophils were placed in GM-CSF and IL-4 for PMN-DC differentiation; precursor and immature “band” neutrophils still present after 4 days further differentiated into neutrophils (F: representative images; G: proportions on right) and the proportion of Ly6G^+^ cells (H) increased after 24 hours of culture with GM-CSF and IL-4; as seen in S10 Fig, the proportion of Ly6G^+^ increases with longer incubation.
(EPS)

S10 Fig Tracking differentiation of ER-HoxB8 GMP cells from neutrophils to PMN-DCs. GMP cells were matured into neutrophils, and at this point (Day 0) neutrophils were cultured up to 7 days with GM-CSF and IL-4. (**A**) Representative microscopy images showing that the starting neutrophils (Day 0) became larger and begin forming projections over 7 days. GFP fluorescence indicates that cells are still viable. Far left column show cells that were stained by Hema3 after cells were spun onto slides. Bar = 10 μm. (**B**) Representative plot of CD11b X Ly6G and FSC X SSC of neutrophils through the course of differentiation. (**C**) Quantification of mean FSC and SSC area through the course of differentiation indicating the neutrophils increase in size and complexity as they differentiate. (**D**) Tracking absolute numbers of canonical neutrophils and PMN-DCs in culture wells over 7 days; wells were seeded with 4 x 10^6^ neutrophils at day 0. (**E**) Tracking expression of CD11c on the surface of neutrophils through 7 days of differentiation; data collected on the same cytometer with the same voltage and calibration settings through the course of the experiment. Mean ± SEM shown for C-E.
(EPS)

S11 Fig Direct fungal killing by canonical neutrophil and PMN-DCs derived from the ER-HoxB8 cell line. (**A**–**B**) Canonical neutrophils or PMN-DCs (GFP^+^) were incubated with *C*. *albicans* yeast (A) or *B*. *dermatitidis* yeast (B) (as in Fig 6A) for 3 hours. Fungi were stained with Uvitex (Blue) and propidium iodide was added to medium to stain DNA (including NETs and nuclei of dead cells). White arrows indicate phagocytosis (shadows in the GFP cells where yeast are). Gold arrows indicate NETs (long strands of extracellular DNA). (**C**) SEM images of canonical neutrophils or PMN-DCs with *B*. *dermatitidis*, demonstrating that PMN-DCs regularly phagocytose yeast while canonical neutrophils rarely phagocytose *B*. *dermatitidis*. (**D**) Filamentation of *C*. *albicans*; plantonic yeast were incubated at 37°C in RPMI plus 2% FBS for up to 2 hours.
(EPS)

S12 Fig Tracking T cell activation by CD44 and CD62L in draining lymph nodes after delivery of vehicle control or antigen-pulsed presenting cells (APCs) subcutaneously. (**A**) Activation of exogenous Tg1807 cells (CD90.1^+^, CD90.2^-^, CD4^+^, CD8α^-^). (**B**–**C**) Activation of endogenous (CD90.1^-^, CD90.2^+^) CD4^+^ (**B**) and CD8^+^ (**C**) T cells showing no effect of antigen pulsing or APC delivery on endogenous T cells indicating the response seen in A is antigen specific. Mean±SEM shown; each point is single mouse; same mice shown in A-C.
(EPS)

S13 Fig Replicate experiment of delivering antigen-pulsed APCs subcutaneously. (**A**) Percent of Tg1807 cell that were activated as indicated by high CD44 and low CD62L expression. (**B**) Absolute number of Tg1807 cells and those activated. This replicate experiment reproduces the effect that antigen-pulsed PMN-DCs increases activation and number of antigen specific T cells but indicates that PMN-DC priming of T cells is comparable and not necessarily better than BMDCs. Statistics indicate significant differences between antigen-pulsed PMN-DC treatment and no antigen controls.
(EPS)

S14 Fig Cytokine products after *in vitro* culture of APCs and Tg1807 cells. (**A**) Presence of IL-6 in supernatants after culture of calnexin or killed *B*. *dermatitidis*, APCs and Tg1807 cells (as in Fig 6F). (**B**) Production of IFN-γ and IL-17 by enriched Tg1807 cells without added BMDCs or PMN-DCs. Recombinant calnexin did stimulate some IFN-γ production likely because a small number of DCs were present after enrichment of spleen and lymph nodes. (**C**) IFN-γ and IL-17 present in from PMN-DCs and BMDCs cultures without T cells but stimulated with calnexin overnight. These cells did produce IL-6 (see Fig 6A) but the amounts of IFN-γ and IL-17 were at or below the lowest standard of 31 pg/ml for IFN-γ and 16 pg/ml for IL-17. (**D**) IFN-γ and IL-17 production by BMDCs stimulated with calnexin or killed *B*. *dermatitidis* for 4 days in the absence of Tg1807 cells. Mean ± SEM is shown.
(EPS)

S15 Fig Role of PMN-DCs in antifungal immunity, modeling PMN-DCs during pulmonary blastomycosis. (**A**) During pulmonary blastomycosis, a small percentage of neutrophils recruited to the lung differentiate into PMN-DCs having features of both neutrophils and DCs. Less than 2% of neutrophils differentiate into PMN-DCs expressing both CD11c and MHC class II; greater than 90% of neutrophils remain canonical neutrophils (CD11c^-^MHCII^-^). These PMN-DCs contribute to antifungal immunity in two ways: through direct killing (B) and antigen presentation (C). (**B**) Direct killing: PMN-DCs associate with and kill fungal cells more frequently than do canonical neutrophils. PMN-DCs retain important neutrophil mechanisms for killing fungi: ROS and NO production, phagocytosis, and the release of NETs. (**C**) PMN-DCs present fungal antigen to CD4^+^ T cells and promote Th1 and Th17 responses important for antifungal immunity. (**D**) Th1 and Th17 cytokines directly and indirectly promote the killing of fungal cells by phagocytic cells [15]. (**E**) Th1 cytokines IFN-γ and GM-CSF have been implicated in driving PMN-DC differentiation [3]. (**F**) IL-17 produced by Th17 cells are known to promote neutrophil recruitment into the lung [63]; greater neutrophil recruitment correlates with and greater numbers of PMN-DCs (Fig 1).
(EPS)
